# Automated Conditional Screening of Multiple *Escherichia coli* Strains in Parallel Adaptive Fed-Batch Cultivations

**DOI:** 10.3390/bioengineering7040145

**Published:** 2020-11-11

**Authors:** Sebastian Hans, Benjamin Haby, Niels Krausch, Tilman Barz, Peter Neubauer, Mariano Nicolas Cruz-Bournazou

**Affiliations:** 1 Technische Universität Berlin, Institute of Biotechnology, Chair of Bioprocess Engineering, Strasse des 17. Juni 135, 10623 Berlin, Germany; benni.haby@web.de (B.H.); n.krausch@tu-berlin.de (N.K.); peter.neubauer@tu-berlin.de (P.N.); 2 AIT Austrian Institute of Technology GmbH, Giefingasse 2, 1210 Vienna, Austria; tilman.barz@ait.ac.at; 3 DataHow AG, ETH Zürich-HCI, F137, Vladimir-Prelog-Weg 1, 8093 Zurich, Switzerland; n.cruz@datahow.ch

**Keywords:** high-throughput screening, rapid phenotyping, model-based experimental design, *Escherichia coli*, automated bioprocess development

## Abstract

In bioprocess development, the host and the genetic construct for a new biomanufacturing process are selected in the early developmental stages. This decision, made at the screening scale with very limited information about the performance in larger reactors, has a major influence on the efficiency of the final process. To overcome this, scale-down approaches during screenings that show the real cell factory performance at industrial-like conditions are essential. We present a fully automated robotic facility with 24 parallel mini-bioreactors that is operated by a model-based adaptive input design framework for the characterization of clone libraries under scale-down conditions. The cultivation operation strategies are computed and continuously refined based on a macro-kinetic growth model that is continuously re-fitted to the available experimental data. The added value of the approach is demonstrated with 24 parallel fed-batch cultivations in a mini-bioreactor system with eight different *Escherichia coli* strains in triplicate. The 24 fed-batch cultivations were run under the desired conditions, generating sufficient information to define the fastest-growing strain in an environment with oscillating glucose concentrations similar to industrial-scale bioreactors.

## 1. Introduction

Emerging technologies in robotic biolaboratories open new opportunities for both high-throughput (HT) screening and HT bioprocess development. On the screening side, significant progress has been made in terms of cultivation scale (down to femtoliter), parallelization and non-invasive observation, which have been widely reviewed [1,2,3]. The focus of this work is conditional screening, where a reduced number of candidate clones are tested under different conditions with the aim to significantly improve the performance at an industrial scale (e.g., media, pH and temperature profiles, bioreactor heterogeneities, induction and feeding strategies). These factors are known to affect the underlying nonlinear dynamics of the bioprocess and are part of the very complex time-dependent interaction between the bioreactor environment and the cell. This highly nonlinear behavior makes it difficult to predict the effect of changes in the cultivating conditions and is responsible for the high failure rate in scale-up [4,5,6]. In order to overcome these challenges, experiments in conditional screening require highly advanced experimental setups able to: (i) operate as similar as possible to the industrial strategy (e.g., fed-batch or continuous cultivations), (ii) mimic the harsh conditions of industrial-scale bioreactors as closely as possible (e.g., growth limitation; bioreactor heterogeneities) and (iii) generate the maximal amount of information possible about the strain’s phenotype and its complex dynamic interaction with the process. Many experimental strategies in all scales as well as kinetic and computational fluid dynamics (CFD) modeling approaches focus on this challenge [7,8,9].

The technology to perform parallel experiments with advanced operation in fed-batch or continuous mode has recently become available [10,11,12,13]. Mini-bioreactors (MBR) integrated in liquid handling stations (LHS) allow a large number of parallel cultivations while maintaining the properties of benchtop bioreactors [9]. With working volumes of 2–250 mL, geometric similarities to large-scale reactors [14] and high-frequency measurements and analytics, MBRs have been used for process characterizations [10,12,15] and scale-down studies [16] for up to 48 cultivations in parallel. Such robotic facilities with automated cultivation control, sampling and at-line analytic operations are very powerful systems that can accelerate bioprocess development [11,17,18,19], especially in combination with digital solutions for experiment (re-)planning [20,21,22], data acquisition [11,17] and real-time dynamic analysis [23]. The bottleneck is currently the lack of advanced computer-aided tools to plan the experiments, operate the robots and build the necessary models and digital twins for scale-up and advanced process control. Because of limitations of the planning and operation capacity of humans, much too often, robots are on hold, waiting for the next experiment to be planned, experimental campaigns need to be repeated because of failures that were not detected on time and the same feeding strategy is used for clones with different characteristics. These are the main issues we address in the present work.

Initial attempts to solve these challenges have demonstrated the added value of model-based tools in terms of accelerating the development process and increasing robustness during scale-up [24]. Nevertheless, the existing solutions are mostly limited to single-strain applications due to the complexity of the used mechanistic models and the difficulty of identifying the parameters for a large number of strains at the same time [25]. Therefore, screening approaches often use simple black-box models for the microorganisms, which do not allow a detailed comparison of their phenotypes.

This contribution proposes an advanced conditional screening design framework and its integration into an autonomously working robotic facility. To achieve this, (i) a macro-kinetic model of the central carbon flux of *Escherichia coli* is defined that can describe the phenotypes of all clones, (ii) a parameter estimation is carried out to cover a characteristic parameter set that describes the individual phenotype and, (iii) based on the unique models, the process is (re)defined in a dynamic process redesign approach as an adapted modelling framework. By this, we not only gain a robust and accurate prediction of the characteristics of each clone but can also quantify and confidently compare their performances. Finally, the method is applied in an online model calibration framework to adaptively define individual optimal feed start and feeding strategies.

**Figure 1:**
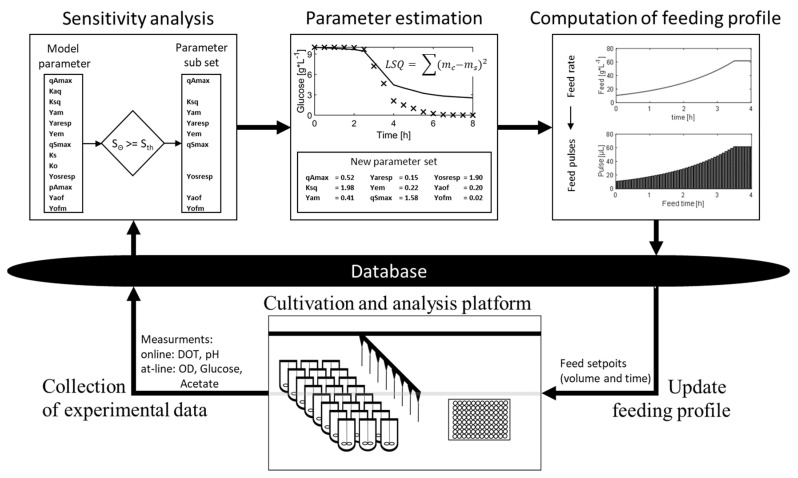
Illustration of the model calibration cycle in the adaptive framework for conditional screening experiments. The cultivation of the clones is performed on the *cultivation and analysis platform* (consisting of two liquid handling stations and a mini-bioreactor system); samples are collected and autonomously analyzed. The generated online and at-line measurements are sent to the central data storage (*database*). The model calibration cycle starts with the collection of all available data. Based on the measurements, the *sensitivity analysis* is performed; based on the results, the identifiable parameters are selected, and non-identifiable parameters are not considered/fixed in the subsequent parameter estimation. In the *parameter estimation*, the identifiable parameter subset is adjusted to fit the model to the measurements. Based on the calibrated model, the feed is calculated during the *feed calculation* step, according to previously defined criteria. The feed is further converted into corresponding pulses with individual times. These time/pulse setpoints are stored in the *database* and executed directly by the *cultivation and analysis platform*.

During the parallel cultivation of this study, the adaptive framework for conditional screening experiments recursively executes the following steps: (i) collection of cultivation data, (ii) selection of an identifiable parameter (sub)set (sensitivity analysis) for each clone, (iii) estimation of kinetic parameters for each clone, (iv) updating of the optimal feeding profiles and (v) transfer of the new feeding profiles to the database (Figure 1). As a proof of concept, parallel screening experiments with eight different clones, including six knockout mutants of *E. coli* K-12, are conducted in 24 mini-bioreactors. At the start of the experiment, virtually no information on the growth behavior of all these strains was available, as it is common in early conditional screening. From the generated data (of all 24 parallel experiments), it was possible to identify 13 model parameters for all clones, with sufficient accuracy to discriminate the performance between the clones.

## 2. Materials and Methods 

### 2.1. HTBD Facility

**Figure 2:**
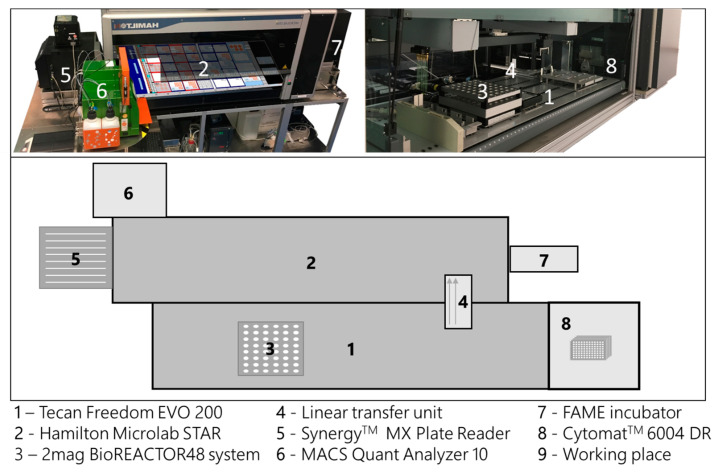
Schematic description of the high-throughput bioprocess development facility used in this study. Composition of the two liquid handling stations (LHS, 1 and 2) and a mini-bioreactor system (3). The liquid handling stations are connected by a linear transfer unit (4) for automatic sample exchange. The facility is surrounded by supporting laboratory equipment (5–8), all accessible by one of the liquid handling stations.

The high-throughput bioprocess development facility is composed of two liquid handling stations (LHS, Freedom Evo 200, Tecan, Switzerland; Microlab Star, Hamilton, Switzerland) and a mini-bioreactor system (48 BioReactor, 2mag AG, Munich, Germany), which is mounted on the Tecan LHS. Both LHSs are connected at the hardware and software level to exchange samples, process and measurement information (Figure 2). The process control (e.g., feed, pH control and volume balance) is carried out by the LHSs in a pulsed based manner. A detailed description of the used hardware and software framework is given in Haby et al. 2019 [17].

### 2.2. Cultivation

Precultures were performed with EnPresso B (Enpresso GmbH, Berlin, Germany) medium with 9 U L^−1^ Reagent A at 37 °C in a 24-multi-well Oxodish plate to keep the cells in the growth phase (PreSens GmbH, Regensburg Germany). The main culture was started as a batch culture at 37 °C with 5 g L^−1^ glucose. The initial batch phase was prolonged after 1 h by an additional feed pulse to a final concentration of 5 g L^−1^ glucose. The stirrer speed was kept constant at 3000 rpm. After the end of the batch phase, a fed-batch was started with a pulse-based glucose feeding every 5 min of feed solution 400 g L^−1^ glucose dissolved in deionized water. The feeding rate was increased exponentially and switched to a constant feed when the maximum pulse volume of 22 µL was reached. In total, the cultivations were carried out over 8 h with fed-batch phases of 5.4 to 6.1 h, depending on the length of the clone-specific batch phases. The µ_set_ for the exponential feed was chosen to be 50% of the model-predicted µ_max_ value and was adapted in every modelling cycle for each clone. The volume of the feed pulses was determined on the basis of the calculated feed rate. All experiments were carried out as biological triplicates.

### 2.3. Sampling and Analytic

During the cultivations, pH and dissolved oxygen tension (DOT) were measured online in the mini-bioreactor system. Each column of the bioreactor system was sampled every 45 min in a sequential mode with a sampling interval of 15 min. Samples were inactivated directly with NaOH and stored in 96-well plates at 4 °C on the deck of the LHS until further processing [20]. After 5 samplings, the sampling plates were automatically transferred to the Hamilton LHS for OD_600_, glucose and acetate measurements in 96-well plates [17]. For the OD_600_ measurements, the samples were diluted to remain in the linear range. The dilution factor was adjusted between 20 and 100 over the course of the cultivation process. All OD_600_ values were multiplied by a correction factor of 2.62 to convert the values to a liquid height of 1 cm. Based on the OD_600_ measurements, the dry cell weight of the biomass was calculated by multiplying the OD_600_ by 0.33 [26]. Due to the time-consuming sampling and analysis procedure, the values for biomass, glucose and acetate were written to the database with a delay of 0.25–1.35 h for the biomass and 0.66–2 h for glucose and acetate, depending on the column of the bioreactor system where the sample was taken.

During the eight hours of cultivation, for each reactor, 1440 values for DOT and pH were collected, as well as 23 samples for biomass (OD_600_), 20 for glucose and 20 for acetate measurements. This yields, in total, for each reactor, 1503 data points. Considering three replicates, the size of the parameter sensitivity matrix is (1503 x 18)*3 (measurements x parameter) * replicates).

### 2.4. Strains

The strains used in this study are *E. coli* K-12 W3110 (F^−^ λ^−^ IN(*rrnD-rrnE*)1 *rph-1*), *E. coli* K-12 BW25113 (F^−^, DE(*araD-araB*)567, *lacZ4787*(del)::*rrnB-3*, LAM^−^, *rph-1*, DE(*rhaD-rhaB*)568, *hsdR514*) and six knockout strains obtained from the NBRP at the National Institute of Genetics, Shizuoka, Japan (Keio collection [27]), namely *E. coli* BW25113-JW0554-KC (*ΔompT*), *E. coli* BW25113-JW3975-KC (*ΔaceA*), *E. coli* BW25133-JW1907-KC (*ΔfliA*), *E. coli* BW25133-JW2076-KC (*ΔgatC*), *E. coli* BW25113-JW2082-KC (*ΔgatZ*) and *E. coli* BW25133-JW2943-KC (*ΔglcB*).

### 2.5. Computational Methods

The *E. coli* macro-kinetic growth model [25] consists of 5 ordinary differential equations, describing biomass, glucose, acetate, oxygen and enzymatic glucose release, and represents the major extracellular dynamics of *E. coli*, including the acetic acid overflow. The model contains 18 parameters, from which 13 have been shown to vary with clones and cultivation conditions. All computational methods related to the model calibration and feed calculation were carried out in MATLAB (The MathWorks, Inc., Natick, Massachusetts, USA), available at https://gitlab.tu-berlin.de/hts_modelling/ModellingFramework. The commit used for this study is efaee5eba813237860264fc33ba79315eef5bbca. Cultivation time and data for the different sequential tasks are summarized in Table 1. All measurements used for the parameter estimation are available in Table S1.

#### 2.5.1. Parameter Estimation

The parameter estimation problem is solved for a reduced (identifiable) parameter subset. This subset is updated in each model calibration cycle in Table 1 and is selected based on the local sensitivity matrix [28]. In doing so, the model calibration updates both the identifiable parameter subset and corresponding parameter values. This approach is useful as the information content in the data increases with each cycle. The algorithm implements a stepwise forward selection of parameters to be included in the estimation problem based on the dynamical parameter sensitivities. Identifiable parameters are selected by a ranking of all parameters according to linear independence and an analysis of the matrix rank condition of the sensitivity matrix.

The parameter estimation is formulated as the following optimization problem:

(1) $$\hat{\theta }≔\underset{\theta }{\mathrm{argmin}}\;\Phi \left(U,\;\theta \right)\;$$

where the objective function reads:

(2) $$\Phi \left(U,\;\theta \right)≔\;\overset{5}{\underset{i=1}{\sum }}\frac{1}{N_{i}}\overset{N_{i}}{\underset{j=1}{\sum }}{\left(y_{i,j}\left(U,\theta \right)-y_{i,j}^{m}\right)}^{2}$$

where $y_{i,j}\left(U,\theta \right)$ are the simulated states and $y_{i,j}^{m}$ are the corresponding measured states. The index i=1,…,5 indicates the measured variables and the index $j=1,\ldots ,N_{i}$ indicates individual data points. 

The CVODES solver in SUNDIALS [29] is used to solve the ODE system and the interior-point algorithm is used for optimisation (MATLAB *fmincon*). Initial values and lower and upper bounds of the parameter estimation (PE) are based on experts’ knowledge and summarized in Table S2. 

#### 2.5.2. Monte Carlo Simulation

Parameter distribution and pairwise correlations are determined by Monte Carlo simulations based on the last dataset (*n* = 500) and with the identifiable parameter set based on the subset selection. Monte Carlo simulations were carried out with σ = 0.15 for biomass, glucose and acetate and with σ = 0.05 for DOT.

#### 2.5.3. Feed Calculation

The exponential feed was calculated using the standard fed-batch equation [30], which was adapted to consider a pulse-based profile. Since the feed in a fed-batch process is the only major volume-changing factor, volume changes due to sampling are neglected at this point and the volume change could be described as

(3) $$\overset{V}{\underset{V_{0}}{\int }}dV=F_{0}\overset{t}{\underset{t_{0}=0}{\int }}e^{{µ}_{set}\cdot t}dt=\frac{F_{0}}{{µ}_{set}}\;e^{{µ}_{set}{\cdot t|}_{0}^{t}}$$

With ${µ}_{set}\;\left[h^{-1}\right]$ as the predefined specific growth rate, $F_{0}\;\left[g\;L^{-1}\right]$ is the initial feed rate and time t [h]. The pulse volume is calculated as

(4) $$V=V_{0}+\frac{F_{0}}{{µ}_{set}}\left(e^{{µ}_{set}\cdot t}-1\right)\;$$

with

(5) F0=µsetYXS * Si X0 V0

where YXS [$g\;g_{x}^{-1}$] is the yield coefficient of glucose per biomass, $S_{i}$ [$g\;L^{-1}$] is the glucose concentration in the feed solution, $X_{0}\;\left[g\right]$ is the biomass concentration and $V_{0}$ is the volume at the feed start. Volume manipulations by the pipetting robot (e.g., volume balancing, sampling and base addition for pH control) are considered in the feed calculation apart from the equations above. 

Biomass and volume for the calculation of $F_{0}$ (Equation (5)) were estimated by simulations based on the last calibrated model. The end of the batch phase was defined as the time point where the predicted glucose and acetate concentrations were below 0.02 g L^−1^, but not later than 45 min after the depletion of glucose.

## 3. Results

Eight different *E. coli* K-12 clones were cultivated in parallel with an industrial process-relevant feeding design consisting of batch, exponential fed-batch and constant feed phases. The feed was applied as pulses to expose the cells to inhomogeneities similar to those in large-scale bioreactors.

### 3.1. Parallel Cultivation

**Figure 3:**
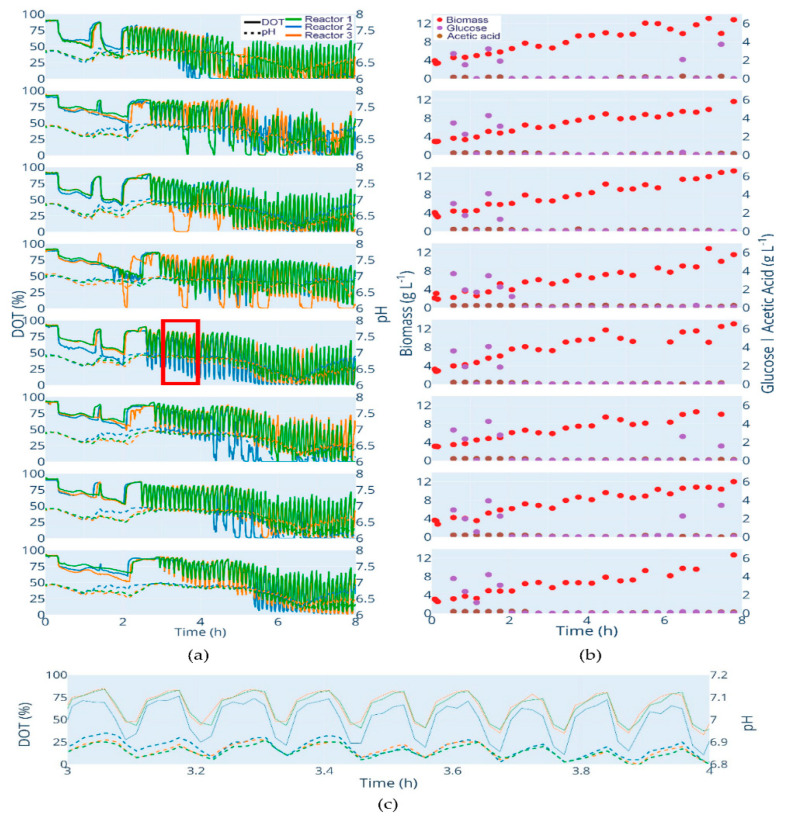
Cultivation data of all strains. Clones from top to bottom: *E. coli* W3110; *E. coli* BW25113; *E. coli* BW25113 *ΔompT*; *E. coli* BW25113 *ΔaceA*; *E. coli* BW25113 *ΔfliA*; *E. coli* BW25113 *ΔgatC*; *E. coli* BW25113 *ΔgatZ*; *E. coli* BW25113 *ΔglcB.* (**a**) DOT (%): solid lines, pH: dotted lines. (**b**) Biomass (g L^−1^): red dots; glucose (g L^−1^): purple dots; acetic acid (g L^−1^): brown dot. (**c**) Illustration of the oscillating pH values with each glucose pulse. The figure shows the section marked in (**a**) red. An interactive version of (**a**,**b**) is available at http://www.bioprocess.tu-berlin.de/fileadmin/fg187/Publications/Hans_2020/fig2.html.

The length of the batch phase varied among the clones and lasted 1.65 h for *E. coli* W3110 (the fastest growing clone) and 1.86 h for *E. coli* BW25113 *ΔglcB* (the slowest growing clone). After the end of the batch phase, the feed was automatically started. Due to the pulse nature of the feed procedure, the feed start is visible through the oscillating DOT values (Figure 3a). These oscillations, as well as the glucose at-line data, prove that glucose limitation was maintained during the fed-batch phase in all cultivations. Furthermore, no significant acetate accumulation was observed (Figure 3b). The cultivations show a low variance between triplicates, which is obvious from the online DOT and pH profiles as well as from the automatically analyzed biomass, glucose and acetate values. As expected, the pH decreased during the batch phase and started to increase after glucose depletion (typically caused by acetate consumption). During each glucose pulse cycle, a perturbation of pH is observed, which is caused by the transient production of acetic acid (Figure 3c). Finally, a small increase in pH was observed after the switch to constant pulse-based feed.

### 3.2. Prediction of Batch and Feed Start

**Figure 4:**
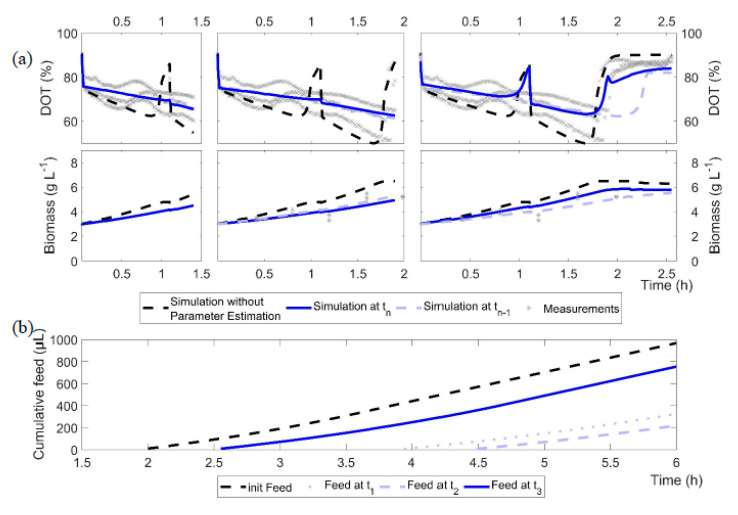
Illustration of the results of the model calibration cycles 1 to 3 for the cultures of *E. coli* BW25113 *ΔglcB*. (**a**) Black dashed line: initial model without adjustment; blue line: current model calibration at time t_n_; light blue line: previous model calibration (time t_n−1_); grey crosses: measurements. (**b**) Computed feeding profiles after model calibration cycles 1 to 3 and the initial feed as a cumulative volume.

After inoculation, samples for biomass were taken and, together with the initial parameter set, served as the basis for the first prediction of the batch phase, feed start and feed rate (Figure 4a, black dashed line). The difference in the initial biomass between the clones led to different simulation results and different predicted batch phases prior to the first model calibration. After the second biomass measurement, the first model calibration cycle was initiated after 1.4 h of batch cultivation and the feed start and rate were re-computed using the updated models. From that time onwards, the model calibration cycle was started after each entry of new at-line data (biomass or glucose/acetate). The end of batch was defined as the time point at which glucose as well as acetate (produced during overflow growth) were depleted (Table 2). Therefore, the fed-batch phase in our cultivations started purposely later compared to typical fed-batch processes, which are mostly started when glucose is depleted and the DO signal increases. Note that feeding was started only when acetate had been metabolized. This prevent possible overfeeding with glucose by co-metabolism of the remaining acetate and thus allowed a higher process stability. 

Figure 4a illustrates the outcome of the model calibration cycle during the batch phase, with the example of the *E. coli* BW25113 *ΔglcB* cultivation data (grey cross) and simulations after model calibration (blue line). It is obvious that the first parameter estimate indicates, for this strain, a slower growth compared to the initial parameter set. However, with each model calibration cycle, the computed growth rate (µ_max_) increased from 0.36 h^−1^ at t_1_ to 0.58 h^−1^ at t_2_ and up to 0.82 h^−1^ at the third shown model calibration cycle. The fit to the cultivation data is improved with each modelling cycle and the trend of the cultivation is well represented, at least after the third modelling cycle.

In addition, due to the underestimated µ_max_, the first model calibration cycle failed to propose the end of the batch phase properly. An accurate estimation of the specific glucose uptake rate is only reached after the first glucose measurements are available, but then the estimation is very precise. Although the glucose depletion is equally estimated in the third model calibration cycle (1.94 h) and in the initial, unadjusted model (1.92 h; Figure 4a, black dashed line), the feed (Figure 4b) started 21 min later (calibrated model: 2.40 h; initial model: 2.01 h). This is due to differences in the production and consumption rates of acetate, resulting in different starting times of the fed-batch phase. Based on the DOT profiles, acetate is consumed after 2.5 h; this also corresponds well with the at-line measurements of *E. coli* BW25113 *ΔglcB* in Figure 3.

The predicted end of the batch phase is very close to the observed one in all cultivations, even after the second model calibration and 1.5 h of cultivation (Table 2). For some cultivations, the time of glucose depletion was predicted with an accuracy of less than one minute (*E. coli* BW25113 *ΔgatZ*). In the worst case, the glucose depletion was predicted 22.8 min too late (*E. coli* BW25113 *ΔaceA*). A missed batch end and even a short starvation phase could lead to unwanted metabolic reactions by the clone and influence the process and product quality. However, with this triplicate, the variance of glucose uptake is very high, because with one triplicate, the glucose consumption is clearly leading (Figure 3a). Without the leading one, the gap between predicted and observed glucose depletion is significant lower. Nevertheless, a difference of 22 min is still in an operational range for conditional screening. Due to operational reasons, the model calibration with all clones was maintained. The overall mean difference between the observed and predicted glucose depletion is 6.9 min for the calibrated model after 1.5 h and, thus, better, compared to the initial model with a mean prediction error of 7.3 min.

Complete consumption of acetate was only observed for five of the eight clones. For all these clones, the adjusted model predicted the acetic acid consumption to better compare to the initial model, with the exception of *E. coli* BW25113 *Δomp*. Complete consumption of acetic acid was not observed for three clones because of the time-dependent restrictions in the feed start (maximum tolerance between end of glucose depletion and feed start; see Section 2.5.3). Nevertheless, for these three clones, the initial model predicted a faster, and the adjusted model, a slower, acetic acid consumption rate. The times of the first feed pulse are summarized in Table 2 (feed start); the predicted end of batch and the first pulse may differ due to technical reasons (delay in computation or first pulses are calculated with 0 µL due to the minimal pipetting volume restrictions).

### 3.3. Feed and Fed-Batch 

**Figure 5:**
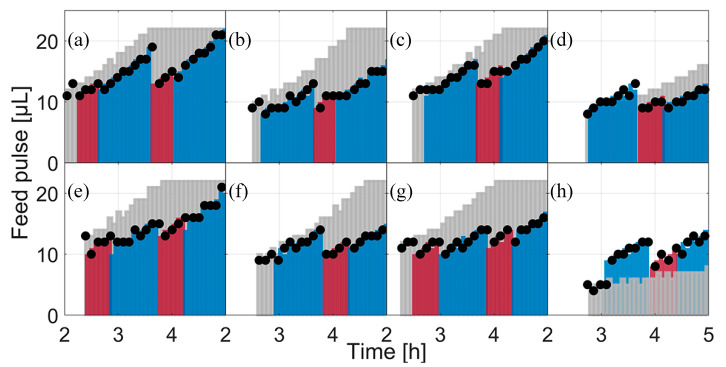
The applied feed and scheduled feeds for each clone between cultivation hours 2 and 5. Black dots: the applied feed based on the real executed feed, logged by the LHS. Light grey bars: scheduled feed at feed start. Colored bars: scheduled feeds in the following modelling cycles; the color change indicates the next modelling cycle. (**a**) *E. coli* W3110; (**b**) *E. coli* BW25113; (**c**) *E. coli* BW25113 *ΔompT*; (**d**) *E. coli* BW25113 *ΔaceA*; (**e**) *E. coli* BW25113 *ΔfliA*; (**f**) *E. coli* BW25113 *ΔgatC*; (**g**) *E. coli* BW25113 *ΔgatZ*; (**h**) *E. coli* BW25113 *ΔglcB.*

During the fed-batch phase, the size of the feed pulses is re-computed during each model calibration cycle. The maximal glucose uptake rate was determined as basis for the new feed rates. However, the biomass concentration and the substrate yield coefficient ($Y_{X/S}$) have a major impact on the initial feed rate ($F_{0}$) and influence the feed as well. With the exception of *E. coli* BW25113 *ΔglcB* (Figure 5h), the first feed rate (grey bars) was higher than the following calculated feed pulses. However, the second applied feed rates for *E. coli* BW25113 *ΔompT*, *E. coli* BW25113 *ΔfliA* and *E. coli* BW25113 *ΔgatZ* (Figure 5c,d,f) were close to the initial feed rates, indicating only a minor parameter drift, but were reduced in the later model calibration cycles. In the case of *E. coli* BW25113 *ΔglcB,* the second feed was somewhat higher than the later one, which is reflected in both the initial feed rate and in the slope of the feed (all feed pulses are summarized in Figure 5). Feed pulses were calculated by the optimization algorithm for each clone and applied to all biological triplicates. In this way, eight different feeding rates were calculated and 24 cultivations were carried out in parallel. 

### 3.4. Parameter Estimation

**Figure 6:**
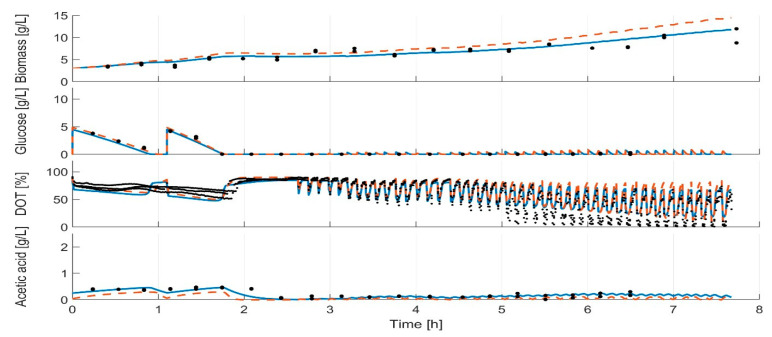
Model for *E. coli* BW25113 *ΔglcB* after the last model calibration cycle and at the beginning of the experiment. Solid line: calibrated model; dashed line: initial model; dots: measurements.

**Figure 7:**
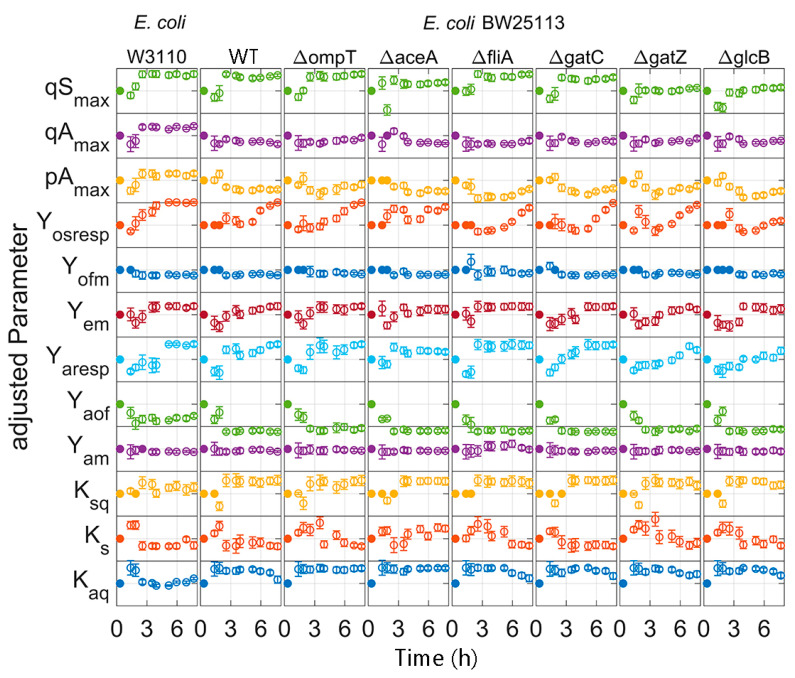
Adjusted parameters of the time for each *E. coli* strain: Parameters are normalized to the initial value and scaled to three times the standard derivation. Filled dots: parameter fixed by the subset selection; open dots: estimated parameter.

During all model calibration cycles, the model parameters are estimated on the basis of all available data (Table S1), i.e., all data which were collected from the start of the cultivations to the actual time point. For all clones, the measurements and dynamics of cultivation are well represented in the simulation of the calibrated model, as illustrated in Figure 6 for the strain *E. coli* BW25113 *ΔglcB* (last modelling cycle; for the other strains, see Supplementary Figures S1–S7). In contrast to the calibrated model, the initial model overestimated the biomass formation. This trend could be observed for all strains, with the exception for *E. coli* BW25113 (Figure S2). The DOT measurements indicate a slower glucose uptake rate than predicted in the last modelling cycle. A lower maximal specific glucose uptake rate (qS_max_) was calculated in the first two modelling calibration cycles compared to the later ones (Figure 7). In the first two model calibration cycles, no glucose measurements were available due to the time delay in the at-line analytics. The prediction error of acetic acid was decreased in the batch and fed-batch phase after model calibration. The cultivation dynamics of all cultivation measurements are well represented by the calibrated model. The parameters to be adjusted in each model calibration cycle are selected by the included subset selection (sensitivity analysis, Figure 1). The parameters K_ap,_ k_L_a and q_m_ are not adjusted in one model calibration cycle. This means that the underlying measurement data are not sufficient to determine these model parameters with sufficient certainty, not even in the last model calibration cycle. The model parameters K_o_, K_sq_, Y_ofm_ and Y_oresp_ are only partly selected for parameter estimation (Figure 7, filed dots). Within the subset selection model parameters can influence other’s parameter sensitivity if they have dependencies to each other. Accordingly, the selection of one parameter may lead to a lower sensitivity of another one and the latter may be excluded from the subset selection although it was active in previous cycles. All parameter subsets and parameter values are summarized in the Supplementary Tables S3–S10. Regularization of parameter estimation using a subsets selection method [28] was used to ensure a meaningful parameter set and to avoid non-physiological model calibrations. 

**Figure 8:**
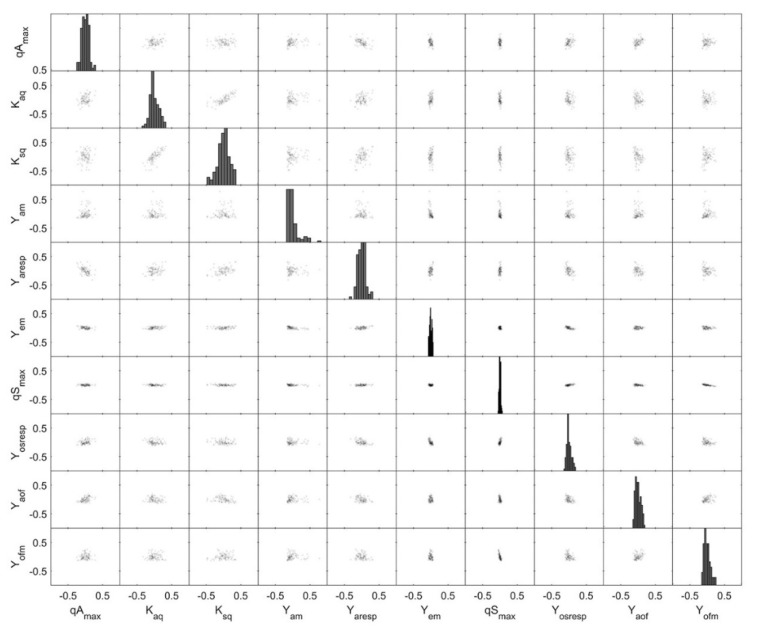
Monte Carlo parameter estimation: pair plots of the 500 best Monte Carlo parameter estimation results and with the identifiable parameter set based on the dataset for *E. coli* BW25113 *ΔglcB* during the last modelling cycle. Monte Carlo simulations were carried out with σ = 0.15 for biomass, glucose and acetate and with σ = 0.05 for DOT.

Monte Carlo simulations have been shown to give a good insight into actual, non-linear parameter distribution [31] and were therefore performed to gain a better understanding of the parameter correlation and its variances. In Figure 8, the parameter distributions for *E. coli* BW25113 *ΔglcB* (last model calibration cycle) are shown based on Monte Carlo simulations. The correlation between all parameters is very weak. Only K_aq_ and K_sq_ showed a correlation with qA_max_. The model parameters K_aq_ and K_sq_ are the affinity constants for acetate and glucose uptake, respectively, and a dependence to the maximal acetate uptake rate (qA_max_) cannot be avoided in the model. The high significance of each parameter is indicated by a narrow distribution in Figure 8 as well as low variation for the most important model parameters (Table 3), especially for the model parameters Y_em_, qS_max_ and Y_osresp._ Normal distribution is given for all parameters except for Y_am_. This parameter is quite close to the lower bound of the previously defined solution space (lower and upper parameter bound). It is noted that this situation should be avoided as it might reduce the accuracy of the parameter estimates.

In the present work, eight strains were examined in 24 successful cultivations. The end of glucose uptake was, in part, predicted with small errors of less than one minute, thanks to the iterative model calibration cycle. The feed start was automatic and in an operable acceptable time window using the dynamic process redesign as defined in the model calibration cycle. The model parameter sets estimated are always unique and with a physiological meaning, even with very little data in the initial phase of this study, e.g., the first 3 h of cultivation. This is ensured by the built-in subset selection and is proven by the Monte Carlo simulations made afterwards.

## 4. Discussion

**Figure 9:**
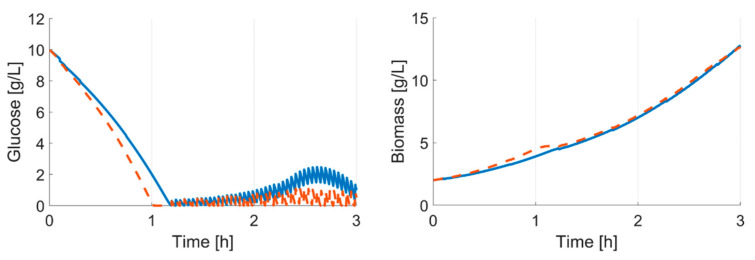
In-silico comparison of different clones; simulation based on last modelling cycle parameter set. Initial values: glucose 10 g L^−1^; biomass 2 g L^-1^. Solid line: *E. coli* BW25113; dashed line: *E. coli* BW25113 *ΔgatZ*. Feed start is simulated at 2.3 h; µ_set_ is fixed at 0.5 h^−1^. If the feed start and rate are only adjusted to one strain, the cultivation of the *ΔgatZ* mutant would lead to overfeeding.

In this study, we presented a computational framework able to design and operate parallel *E. coli* cultivations without human supervision. The results demonstrate that a robust operation tailored to each specific clone is possible through an adaptive input design. Undesired experimental conditions (e.g., overfeeding and starvation) are avoided while sufficient information to allow a confident discrimination of the clones is generated. Both start time and feed rate were accurately predicted for each one of the eight clones, using feedback information from online and at-line cultivation measurements. This is essential in an experimental facility aimed to perform screening cultivations for clones whose phenotype is not known beforehand. The relevance of an adaptive and specific experimental design can be seen in this case study. As illustrated in Figure 9, despite the fact that the clone characteristics differ only minimally from each other, an experiment with a fixed start time and feeding rate would have violated important experimental constrains (here, overfeeding). 

Additionally, the use of a macro-kinetic growth model that describes the main extracellular dynamics of *E. coli* was shown to be sufficient, even though it is insufficient to describe the complex nonlinear dynamics of the bioprocess and the different genotypes of the clones. The adaptive nature of the framework ensures a proper prediction within the current horizon and is sufficient to assure a robust operation of the cultivations. On average, the predicted feed start differed by less than 10 min for the optimal one, which is in an acceptable range and is mainly caused by unobserved disturbances in the system. If necessary, the mismatch can be further reduced by increasing the frequency of model calibration. Furthermore, the framework provides all necessary parameters and actions to define a wide range of alternative feet start triggers (e.g., glucose reduction or acetate consumption).

**Figure 10:**
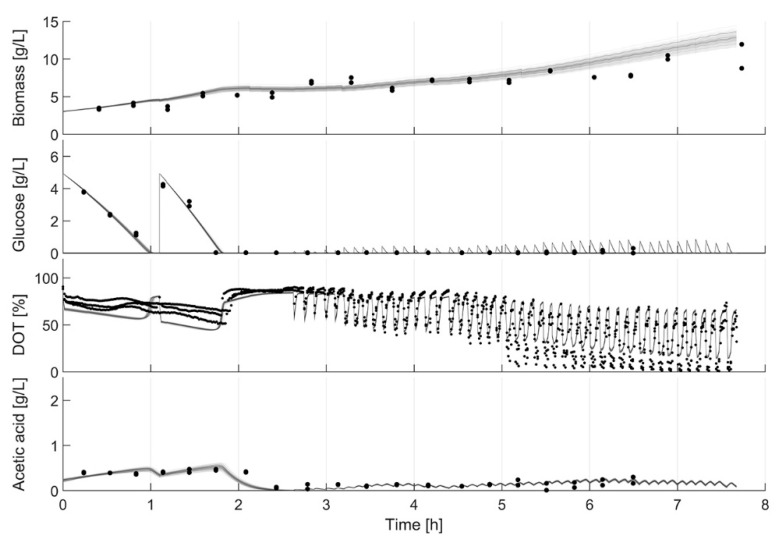
Model uncertainty based on parameter standard deviation. Monte Carlo simulation: results of 1000 parameter estimates based on in-silico data. In-silico data were generated based on the last data set for *E. coli* BW25113 *ΔglcB* and by a random σ of 0.15 for biomass, glucose and acetate and a σ of 0.05 for DOT.

As expected, the parameter variance in general decreases with every model calibration cycle. After the cultivation, the parameter distribution is generally very narrow. This is also reflected in the small deviations of the simulation results of the Monte Carlo studies (Figure 10) and demonstrates the value of the parameters for further in-silico studies. Important parameters, such as maximum glucose uptake (qS_max_), can be distinguished with statistical significance and used for clone discrimination (Figure 7, Table 3). In this study, *E. coli* BW25113 ∆*ompT* had the largest qS_max_ value and *E. coli* BW25113 ∆*gatZ* the lowest one. However, some parameters could not, or only with insufficient confidence, be identified. This hampered a distinction of some essential parameters, such as the maximal acetate uptake rate (qA_max_). Furthermore, parameter identifiability can be increased in future applications using methods for optimal experimental design (OED) [20,32,33] or enhanced parameter identifiability analysis [34,35,36]. 

The frequency of the parameter estimation was defined based on the availability of at-line data (biomass, glucose and acetate), and as expected, the at-line data are decisive to achieve model identifiability. Still, the results show that especially parameters related to glucose consumption can be identified using only the online DOT signal. This shows that, even though in a significantly limited manner, the framework can also be used to increase the robustness of robotic facilities that do not have embedded at-line analytics. This significantly reduces the operative effort of the experimental setup. The glucose consumption rate seems to be observable from the DOT signal, by which a reduced version of the macro-kinetic model could be used to build an observer-based feeding control. Finally, we also demonstrated that the length of the batch phase is essential to assure sufficient data before the start of the feeding so as to allow a reliable operation of the following phases. 

Some parameters drift over time or change rapidly between batch and fed-batch (Figure 7). This could be related to the increasing information content of the growing data set and the resulting addition of previously neglected model parameters to the subset selection. However, the variations in the parameters caused by intracellular changes in the metabolic machinery together with heterogeneous mutations in the population [37] are not represented in the model and could also cause such parameter changes. Such uncovered intracellular changes may also explain the apparently poor representation of the batch phase in the last model calibration cycle compared to earlier ones. Therefore, an iterative recalculation of the feed is necessary to cope with disturbances in the experiments and inaccuracies in the model prediction. Moving horizon approaches can also increase the model prediction accuracy by allowing different parameter sets in different cultivation phases for a single clone [38].

## 5. Conclusions

The operation of robotic experiments with multiple fed-batch cultivations in parallel is very challenging even for skilled operators, since many decisions and tasks are needed at the same time. In this work, we present an adaptive framework for conditional screening for parallel fed-batch experiments, aiming to identify the best candidate strain for industrial scale biomanufacturing. We demonstrate that the use of a macro-kinetic growth model in an adaptive framework using online and at-line data information in a feedback loop is necessary to:

1. Design a specific strategy for each different clone of the conditional screening experiment.
2. Increase the robustness of the robotic operation against experimental disturbance.
3. Give an approximation of the reliability of the simulation results with respect to production scale performance.

To our knowledge, this is the first successful model-based operation of 24 fed-batch cultivations with as many as eight different clones in parallel including its characterization, sufficient for clone discrimination. The results clearly demonstrate the capabilities of the framework to increase the efficiency of combined mini-bioreactor systems with liquid handling stations to drastically reduce the experimental time, efforts and failure rate in high-throughput bioprocess development. 

## Figures and Tables

**Table 1:** Underlying data sets, i.e., the number of sensor data, for the parameter estimates of one biological triplicate. DOT—dissolved oxygen tension.

Sequential Task (Iteration)	Cultivation Time (h)	Available Measurements			
		DOT	Biomass	Glucose	Acetate
1	1.38	321	6	0	0
2	1.88	411	16	0	0
3	2.55	531	16	10	10
4	3.52	705	26	10	10
5	3.93	780	26	20	20
6	5.17	999	36	20	20
7	5.94	1137	36	30	30
8	6.91	1311	46	30	30
9	7.66	1440	46	40	40

**Table 2:** Batch end prediction overview: initial model, adjusted model (parameter estimation after 1.88 h) and observed times for consumption of glucose and acetate and the actual feed start based on the first executed glucose pulse.

Strain	Glucose Consumption (hh:mm)			Acetate Consumption (hh:mm)			Feed Start (hh:mm)
	Initial	Adjusted	Observed	Initial	Adjusted	Observed	
*E. coli* W3110	01:46	01:40	01:39 ± 00:01	02:03	01:48	01:48	01:55
*E. coli* BW25113	01:52	01:38	01:49 ± 00:03	02:00	03:02	>02:23	02:23
*E. coli* BW25113 *ΔompT*	01:46	01:40	01:40 ± 00:01	01:53	03:05	02:10	02:23
*E. coli* BW25113 *ΔaceA*	02:13	02:11	01:48 ± 00:21	02:22	03:06	>02:37	02:37
*E. coli* BW25113 *ΔfliA*	01:51	01:36	01:42 ± 00:01	01:59	02:13	02:07	02:16
*E. coli* BW25113 *ΔgatC*	01:49	01:39	01:46 ± 00:05	01:57	02:50	02:25	02:30
*E. coli* BW25113 *ΔgatZ*	01:46	01:43	01:43 ± 00:01	01:55	03:03	>02:09	02:09
*E. coli* BW25113 *ΔglcB*	01:55	01:56	01:51 ± 00:03	02:03	02:24	02:30	02:37

**Table 3:** Values, variance and relative variance of the adjusted parameters for all clones after the final model calibration cycle.

			*E.coli* W3110			*E. coli* BW215113																				
Parameter	Unit	Inital Guess				WT			ΔompT			ΔaceA			ΔfliA			ΔgatC			ΔgatZ			ΔglcB		
			θ	σ_θ_	%σ_θ_	θ	σ_θ_	%σ_θ_	θ	σ_θ_	%σ_θ_	θ	σ_θ_	%σ_θ_	θ	σ_θ_	%σ_θ_	θ	σ_θ_	%σ_θ_	θ	σ_θ_	%σ_θ_	θ	σ_θ_	%σ_θ_
**qAmax**	g g^−1^ h^−1^	1.0252	1.59	0.11	6.78	0.52	0.11	21.74	0.90	0.05	5.92	0.56	0.15	26.05	0.72	0.11	15.59	0.68	0.08	12.19	0.86	0.10	11.54	0.71	0.07	9.54
**Kaq**	g L^−1^	0.2133	0.59	0.14	23.11	0.55	0.09	16.28	0.98	0.07	7.11	0.98	0.14	13.82	0.60	0.12	20.70	0.68	0.10	14.16	0.75	0.12	15.97	0.70	0.04	6.24
**Ksq**	g L^−1^	1.0667	1.52	0.33	21.71	1.98	0.34	16.98	1.97	0.26	13.17	1.91	0.32	16.95	1.77	0.29	16.26	1.99	0.25	12.35	1.63	0.31	18.80	1.68	0.34	20.14
**Yam**	g g^−1^	0.1955	0.40	0.03	7.18	0.41	0.04	10.79	0.44	0.05	10.16	0.44	0.08	18.79	0.48	0.04	8.04	0.44	0.08	17.80	0.44	0.04	8.89	0.42	0.03	6.82
**Yaresp**	g g^−1^	0.1672	0.15	0.01	4.94	0.15	0.01	5.09	0.15	0.01	4.55	0.12	0.01	8.36	0.15	0.01	4.79	0.15	0.01	8.02	0.13	0.01	6.65	0.13	0.00	3.44
**Yem**	g g^−1^	0.56	0.60	0.01	2.48	0.60	0.01	1.82	0.60	0.02	2.89	0.58	0.02	2.70	0.60	0.01	1.94	0.60	0.02	3.27	0.58	0.01	2.19	0.59	0.01	1.53
**qSmax**	g g^−1^ h^−1^	1.3431	1.60	0.02	1.20	1.58	0.03	2.09	1.60	0.03	2.02	1.47	0.03	1.79	1.59	0.03	1.83	1.55	0.03	2.08	1.39	0.02	1.16	1.40	0.04	2.54
**Ks**	g L^−1^	0.05	0.03	0.01	22.73	0.03	0.00	15.72	0.03	0.01	27.93	0.08	0.01	8.44	0.03	0.01	19.59	0.03	0.01	21.88	0.04	0.01	29.35	0.03	0.01	18.79
**Ko**	g L^−1^	1	19.87	1.52	7.64	18.13	1.87	10.32	14.51	1.27	8.76	18.64	1.93	10.38	16.29	1.66	10.17	16.00	2.22	13.88	14.13	0.95	6.69	9.57	1.27	13.28
**Yosresp**	g g^−1^	1	2.00	0.05	2.54	2.00	0.04	1.90	1.99	0.09	4.46	1.80	0.09	4.78	1.76	0.05	3.03	1.97	0.08	4.31	1.89	0.06	3.04	1.19	0.02	2.03
**pAmax**	g g^−1^ h^−1^	1.3091	1.60	0.07	4.18	0.93	0.12	13.29	1.13	0.08	7.31	0.86	0.07	8.64	0.95	0.07	7.45	0.98	0.09	8.96	1.08	0.09	8.37	0.87	0.09	10.77
**Yaof**	g g^−1^	0.4607	0.35	0.01	3.88	0.20	0.02	12.23	0.24	0.02	7.52	0.21	0.02	6.99	0.21	0.02	7.05	0.20	0.02	8.88	0.23	0.02	8.21	0.23	0.02	7.91
**Yofm**	g g^−1^	0.2795	0.20	0.01	4.74	0.20	0.02	7.53	0.22	0.01	5.46	0.21	0.02	9.87	0.23	0.01	5.75	0.21	0.02	10.21	0.22	0.01	5.20	0.22	0.01	5.08

## Supplementary Materials

The following are available online at https://www.mdpi.com/2306-5354/7/4/145/s1, Table S1: all experimental measurements used for the modelling cycles; Table S2: initial parameter values, upper and lower bounds and units; Table S3: parameter subsets and values for *E. coli* W3110; Table S4: parameter subsets and values for *E. coli* BW25113; Table S5: parameter subsets and values for BW25113 *ΔompT*; Table S6: parameter subsets and values for BW25113 *ΔaceA*; Table S7: parameter subsets and values for BW25113 *ΔfliA*; Table S8: parameter subsets and values for BW25113 *ΔgatC*; Table S9: parameter subsets and values for BW25113 *ΔgatZ*; Table S10: parameter subsets and values for BW25113 *ΔglcB*; Figure S1: Comparison of measurement and simulation for *E. coli* W3110; Figure S2: Comparison of measurement and simulation for *E. coli* BW25113; Figure S3: Comparison of measurement and simulation for *E. coli* BW25113 *ΔompT*; Figure S4: Comparison of measurement and simulation for *E. coli* BW25113 *ΔaceA*; Figure S5: Comparison of measurement and simulation for *E. coli* BW25133 *ΔfliA*; Figure S6: Comparison of measurement and simulation for *E. coli* BW25133 *ΔgatC*; Figure S7: Comparison of measurement and simulation for *E. coli* BW25113 *ΔgatZ*.
