# Supplementary material for: Automated Conditional Screening of Multiple Escherichia coli Strains in Parallel Adaptive Fed-Batch Cultivations
Source: Bioengineering (Basel). 2020 Nov 11;7(4):145. doi: 10.3390/bioengineering7040145 (PMC7711848; doi:10.3390/bioengineering7040145)
Supplement: Supplementary file 1 [file bioengineering-07-00145-s001.zip › Hans_2020_Supplementary.pdf]

Automated conditional screening of multiple *Escherichia coli* strains in parallel adaptive fed-batch cultivations

Sebastian Hans [1*], Benjamin Haby [1], Niels Krausch [1], Tilman Barz [2], Peter Neubauer [1] and Mariano Nicolas Cruz-Bournazou[3]

[1] Technische Universität Berlin, Faculty III Process Sciences, Institute of Biotechnology, Chair of Bioprocess Engineering, Ackerstraße 76, 13357 Berlin, Germany; peter.neubauer@tu-berlin.de
[2] AIT Austrian Institute of Technology, Center for Energy; tilman.barz@ait.ac.at
[3] DataHow AG, ETH Zürich-HCI, F137, Vladimir-Prelog-Weg 1, 8093 Zurich, Switzerland; n.cruz@datahow.ch

* Correspondence: sebastian.hans@tu-berlin.de

Supplementary Table S1: Experimental measurements

For raw data and further explanations see File: Table_S1_Measurments.xlsx

Supplementary Table S2: Initial parameter values and bounds for the parameter estimation (model calibration):

Parameter	Unit	Lower Bound	initial Value	Upper Bound
K_{ap}	g L^{-1}	1.00	4.44	10.00
	Monod-type saturation constant, intracellular acetate production			
K_{aq}	g L^{-1}	0.30	0.40	1.00
	Affinity constant, acetate consumption			
k_La	h^{-1}	200.00	280.00	300.00
	Volume specific mass transfer coefficient			
K_o	g L^{-1}	1.00	10.00	20.00
	Affinity constant, oxygen consumption			
K_s	g L^{-1}	0.03	0.05	0.10
	Affinity constant, substrate consumption			
K_{sq}	g L^{-1}	0.10	1.07	2.00
	Inhibition constant, inhibition of acetate uptake by glucose			
pA_{max}	g g^{-1} h^{-1}	0.50	1.31	1.60
	Maximal specific acetate production rate			
$qA_{ma}x$	g g^{-1} h^{-1}	0.50	1.03	1.60
	Maximal specific acetate uptake rate			
q_m	g g^{-1} h^{-1}	0.02	0.04	0.05
	Specific maintenance coefficient			
qS_{max}	g g^{-1} h^{-1}	1.00	1.56	1.60
	Maximal specific glucose uptake rate			
Y_{am}	g g^{-1}	0.40	0.40	1.00
	Yield of acetate to biomass			
Y_{aof}	g g^{-1}	0.20	0.46	0.60
	Yield of glucose uptake to overflow metabolism			

Parameter	Unit	Lower Bound	initial Value	Upper Bound
Y_{aresp}	$g\ g^{-1}$	0.05	0.10	0.15
	Yield of acetate uptake to oxygen consumption			
Y_{em}	$g\ g^{-1}$	0.50	0.56	0.60
	Yield of glucose to biomass			
Y_{ofm}	$g\ g^{-1}$	0.20	0.28	0.80
	Yield of overflow metabolism to biomass			
Y_{osresp}	$g\ g^{-1}$	0.70	1.00	2.00
	Yield of glucose uptake to oxygen consumption			

Supplementary Table S3: Parameter values after each model calibration (MC) for *Escherichia coli* K12 W3110. Parameters marked with * are adjusted in the MC

	MC 1		MC 2		MC 3		MC 4		MC 5		MC 6		MC 7		MC 8		MC 9	
**Parameter**	SSS	Par	SSS	Par	SSS	Par	SSS	Par	SSS	Par	SSS	Par	SSS	Par	SSS	Par	SSS	Par
K_{ap}		4.44		4.44		4.44		4.44		4.44		4.44		4.44		4.44		4.44
K_{aq}	*	0.99	*	0.90	*	0.46	*	0.41	*	0.32	*	0.32	*	0.45	*	0.44	*	0.59
K_o		10.00		10.00		10.00		10.00		10.00	*	2.67	*	16.17	*	16.47	*	19.87
K_s	*	0.08	*	0.08	*	0.03	*	0.03	*	0.03	*	0.03	*	0.03	*	0.05	*	0.03
K_{sq}	*	1.25		1.07	*	1.82	*	1.71	*	1.10	*	1.48	*	1.60	*	1.33	*	1.52
Y_{am}	*	0.41	*	0.46		0.50	*	0.40	*	0.40	*	0.42	*	0.41	*	0.42	*	0.40
Y_{aof}	*	0.38	*	0.28	*	0.32	*	0.33	*	0.30	*	0.31	*	0.33	*	0.33	*	0.35
Y_{aresp}	*	0.06	*	0.07	*	0.09	*	0.08	*	0.08	*	0.15	*	0.15	*	0.15	*	0.15
Y_{em}	*	0.56	*	0.52	*	0.55	*	0.60	*	0.60	*	0.60	*	0.60	*	0.59	*	0.60
Y_{ofm}		0.28	*	0.23	*	0.20	*	0.20	*	0.20	*	0.21	*	0.20	*	0.21	*	0.20
Y_{osresp}	*	0.73	*	1.08	*	1.44	*	1.59	*	1.86	*	2.00	*	2.00	*	1.98	*	2.00
k_La		280.00		280.00		280.00		280.00		280.00		280.00		280.00		280.00		280.00
pA_{max}	*	0.88	*	1.12	*	1.58	*	1.59	*	1.48	*	1.59	*	1.60	*	1.50	*	1.60
qA_{max}	*	0.51	*	0.70	*	1.53	*	1.55	*	1.52	*	1.40	*	1.55	*	1.45	*	1.59
qS_{max}	*	1.27	*	1.41	*	1.59	*	1.60	*	1.60	*	1.58	*	1.60	*	1.57	*	1.60
q_m		0.04		0.04		0.04		0.04		0.04		0.04		0.04		0.04		0.04

Supplementary Table S4: Parameter values after each model calibration (MC) for *Escherichia coli* K12 BW25113. Parameters marked with * are adjusted in the MC *

	MC 1		MC 2		MC 3		MC 4		MC 5		MC 6		MC 7		MC 8		MC 9	
**Parameter**	SSS	Par	SSS	Par	SSS	Par	SSS	Par	SSS	Par	SSS	Par	SSS	Par	SSS	Par	SSS	Par
K_{ap}		4.44		4.44		4.44		4.44		4.44		4.44		4.44		4.44		4.44
K_{aq}	*	0.95	*	0.98	*	0.88	*	0.88	*	0.93	*	0.93	*	0.86	*	0.81	*	0.55
K_o		10.00		10.00		10.00		10.00		10.00		10.00	*	17.13	*	18.26	*	18.13
K_s	*	0.06	*	0.08	*	0.03	*	0.03	*	0.04	*	0.04	*	0.04	*	0.03	*	0.03
K_{sq}		1.07	*	0.21	*	1.97	*	1.97	*	1.90	*	1.91	*	1.82	*	1.95	*	1.98
Y_{am}	*	0.51	*	0.42	*	0.41	*	0.42	*	0.44	*	0.47	*	0.48	*	0.45	*	0.41
Y_{aof}	*	0.32	*	0.38	*	0.20	*	0.20	*	0.21	*	0.21	*	0.22	*	0.20	*	0.20
Y_{aresp}	*	0.06	*	0.06	*	0.13	*	0.13	*	0.11	*	0.12	*	0.13	*	0.15	*	0.15
Y_{em}	*	0.52	*	0.50	*	0.55	*	0.58	*	0.56	*	0.58	*	0.59	*	0.60	*	0.60
Y_{ofm}		0.28		0.28	*	0.20	*	0.20	*	0.21	*	0.22	*	0.23	*	0.21	*	0.20
Y_{osresp}		1.00		1.00	*	1.30	*	1.19	*	1.04	*	1.14	*	1.64	*	1.86	*	2.00
k_La		280.00		280.00		280.00		280.00		280.00		280.00		280.00		280.00		280.00
pA_{max}	*	1.32	*	1.57	*	1.03	*	0.94	*	0.89	*	0.89	*	0.97	*	0.92	*	0.93
qA_{max}	*	0.57	*	0.53	*	0.82	*	0.73	*	0.65	*	0.66	*	0.69	*	0.62	*	0.52
qS_{max}	*	1.25	*	1.31	*	1.59	*	1.57	*	1.55	*	1.53	*	1.54	*	1.56	*	1.58
q_m		0.04		0.04		0.04		0.04		0.04		0.04		0.04		0.04		0.04

Supplementary Table S5: Parameter values after each model calibration (MC) for *Escherichia coli* K12 BW25113 *ΔompT*. Parameters marked with * are adjusted in the MC *

	MC 1		MC 2		MC 3		MC 4		MC 5		MC 6		MC 7		MC 8		MC 9	
**Parameter**	SSS	Par	SSS	Par	SSS	Par	SSS	Par	SSS	Par	SSS	Par	SSS	Par	SSS	Par	SSS	Par
**K_{ap}**		4.44		4.44		4.44		4.44		4.44		4.44		4.44		4.44		4.44
**K_{aq}**	*	0.94	*	0.95	*	0.93	*	0.98	*	0.96	*	0.91	*	0.91	*	0.97	*	0.98
**K_{o}**		10.00		10.00		10.00		10.00		10.00	*	15.63	*	15.80	*	15.58	*	14.51
**K_{s}**	*	0.07	*	0.08	*	0.07	*	0.09	*	0.04	*	0.06	*	0.04	*	0.03	*	0.03
**K_{sq}**	*	1.09	*	0.41	*	1.74	*	1.83	*	1.90	*	1.49	*	1.70	*	1.92	*	1.97
**Y_{am}**	*	0.45	*	0.44	*	0.48	*	0.44	*	0.44	*	0.49	*	0.47	*	0.46	*	0.44
**Y_{aof}**	*	0.36	*	0.34	*	0.23	*	0.21	*	0.21	*	0.24	*	0.25	*	0.22	*	0.24
**Y_{aresp}**	*	0.07	*	0.07	*	0.12	*	0.15	*	0.14	*	0.12	*	0.13	*	0.15	*	0.15
**Y_{em}**	*	0.55	*	0.52	*	0.57	*	0.60	*	0.60	*	0.59	*	0.58	*	0.60	*	0.60
**Y_{ofm}**		0.28		0.28	*	0.26	*	0.23	*	0.22	*	0.25	*	0.23	*	0.23	*	0.22
**Y_{osresp}**	*	0.82	*	1.08	*	0.88	*	0.94	*	1.16	*	1.29	*	1.61	*	1.89	*	1.99
**$k_{L}a$**		280.00		280.00		280.00		280.00		280.00		280.00		280.00		280.00		280.00
**pA_{max}**	*	1.12	*	1.38	*	0.90	*	0.73	*	0.86	*	0.93	*	1.09	*	1.02	*	1.13
**qA_{max}**	*	0.59	*	0.57	*	0.65	*	0.55	*	0.65	*	0.71	*	0.80	*	0.75	*	0.90
**qS_{max}**	*	1.25	*	1.35	*	1.55	*	1.58	*	1.55	*	1.56	*	1.57	*	1.59	*	1.60
**q_{m}**		0.04		0.04		0.04		0.04		0.04		0.04		0.04		0.04		0.04

Supplementary Table S6: Parameter values after each model calibration (MC) for *Escherichia coli* K12 BW25113 *ΔaceA*. Parameters marked with * are adjusted in the MC *

	**MC 1**		**MC 2**		**MC 3**		**MC 4**		**MC 5**		**MC 6**		**MC 7**		**MC 8**		**MC 9**	
**Parameter**	SSS	Par	SSS	Par	SSS	Par	SSS	Par	SSS	Par	SSS	Par	SSS	Par	SSS	Par	SSS	Par
K_{ap}		4.44		4.44		4.44		4.44		4.44		4.44		4.44		4.44		4.44
K_{aq}	*	1.00	*	0.99	*	0.94	*	0.85	*	0.94	*	0.97	*	0.98	*	0.98	*	0.98
K_o		10.00		10.00		10.00		10.00		10.00		10.00		10.00		10.00	*	18.64
K_s	*	0.07	*	0.07	*	0.03	*	0.04	*	0.06	*	0.07	*	0.06	*	0.08	*	0.08
K_{sq}		1.07	*	0.60		1.07	*	1.92	*	1.93	*	1.91	*	1.94	*	1.94	*	1.91
Y_{am}	*	0.40	*	0.41	*	0.41	*	0.46	*	0.45	*	0.45	*	0.44	*	0.43	*	0.44
Y_{aof}	*	0.32	*	0.33	*	0.21	*	0.22	*	0.21	*	0.21	*	0.21	*	0.21	*	0.21
Y_{aresp}	*	0.09	*	0.08	*	0.14	*	0.13	*	0.12	*	0.13	*	0.13	*	0.13	*	0.12
Y_{em}	*	0.59	*	0.51	*	0.55	*	0.59	*	0.56	*	0.58	*	0.59	*	0.58	*	0.58
Y_{ofm}		0.28		0.28	*	0.21	*	0.26	*	0.21	*	0.21	*	0.21	*	0.21	*	0.21
Y_{osresp}		1.00	*	1.40	*	1.72	*	1.67	*	1.23	*	1.26	*	1.69	*	1.63	*	1.80
k_La		280.00		280.00		280.00		280.00		280.00		280.00		280.00		280.00		280.00
pA_{max}		1.31		1.31	*	1.05	*	1.09	*	0.82	*	0.77	*	0.91	*	0.86	*	0.86
qA_{max}	*	0.51		1.03	*	1.29	*	0.99	*	0.64	*	0.59	*	0.62	*	0.57	*	0.56
qS_{max}	*	1.46	*	1.05	*	1.50	*	1.45	*	1.44	*	1.44	*	1.45	*	1.46	*	1.47
q_m		0.04		0.04		0.04		0.04		0.04		0.04		0.04		0.04		0.04

Supplementary Table S7: Parameter values after each model calibration (MC) for *Escherichia coli* K12 BW25113 *ΔfliA*. Parameters marked with * are adjusted in the MC *

	MC 1		MC 2		MC 3		MC 4		MC 5		MC 6		MC 7		MC 8		MC 9	
**Parameter**	SSS	Par	SSS	Par	SSS	Par	SSS	Par	SSS	Par	SSS	Par	SSS	Par	SSS	Par	SSS	Par
**K_{ap}**		4.44		4.44		4.44		4.44		4.44		4.44		4.44		4.44		4.44
**K_{aq}**	*	0.99	*	0.92	*	0.99	*	0.98	*	0.98	*	0.98	*	0.79	*	0.70	*	0.60
**K_{o}**		10.00		10.00		10.00		10.00		10.00	*	18.53	*	19.82	*	18.77	*	16.29
**K_{s}**	*	0.05	*	0.07	*	0.09	*	0.08	*	0.06	*	0.07	*	0.04	*	0.03	*	0.03
**K_{sq}**		1.07		1.07	*	1.97	*	1.89	*	1.90	*	1.83	*	1.93	*	1.89	*	1.77
**Y_{am}**	*	0.41	*	0.44	*	0.43	*	0.59	*	0.59	*	0.60	*	0.66	*	0.54	*	0.48
**Y_{aof}**	*	0.31	*	0.27	*	0.20	*	0.22	*	0.21	*	0.22	*	0.20	*	0.21	*	0.21
**Y_{aresp}**	*	0.05	*	0.06	*	0.15	*	0.14	*	0.14	*	0.14	*	0.15	*	0.15	*	0.15
**Y_{em}**	*	0.56	*	0.53	*	0.60	*	0.60	*	0.59	*	0.60	*	0.60	*	0.60	*	0.60
**Y_{ofm}**		0.28	*	0.39	*	0.21	*	0.26	*	0.25	*	0.28	*	0.24	*	0.23	*	0.23
**Y_{osresp}**		1.00		1.00	*	0.72	*	0.75	*	0.77	*	0.91	*	1.15	*	1.55	*	1.76
**$k_{L}a$**		280.00		280.00		280.00		280.00		280.00		280.00		280.00		280.00		280.00
**pA_{max}**	*	1.10	*	1.05	*	0.56	*	0.62	*	0.62	*	0.61	*	0.67	*	0.85	*	0.95
**qA_{max}**	*	0.52	*	0.52	*	0.51	*	0.56	*	0.53	*	0.54	*	0.52	*	0.65	*	0.72
**qS_{max}**	*	1.34	*	1.37	*	1.60	*	1.56	*	1.54	*	1.56	*	1.55	*	1.59	*	1.59
**q_{m}**		0.04		0.04		0.04		0.04		0.04		0.04		0.04		0.04		0.04

Supplementary Table S8: Parameter values after each model calibration (MC) for *Escherichia coli* K12 BW25113 *ΔgatC*. Parameters marked with * are adjusted in the MC *

	**MC 1**		**MC 2**		**MC 3**		**MC 4**		**MC 5**		**MC 6**		**MC 7**		**MC 8**		**MC 9**	
**Parameter**	SSS	Par	SSS	Par	SSS	Par	SSS	Par	SSS	Par	SSS	Par	SSS	Par	SSS	Par	SSS	Par
K_{ap}		4.44		4.44		4.44		4.44		4.44		4.44		4.44		4.44		4.44
K_{aq}	*	0.95	*	0.91	*	0.80	*	0.92	*	0.97	*	0.99	*	0.88	*	0.87	*	0.68
K_{o}		10.00		10.00		10.00		10.00		10.00		10.00	*	17.83	*	16.73	*	16.00
K_{s}	*	0.07	*	0.07	*	0.04	*	0.03	*	0.03	*	0.03	*	0.03	*	0.03	*	0.03
K_{sq}		1.07	*	0.41		1.07	*	1.96	*	1.97	*	1.99	*	1.94	*	1.95	*	1.99
Y_{am}	*	0.47	*	0.46	*	0.44	*	0.42	*	0.42	*	0.40	*	0.44	*	0.45	*	0.44
Y_{aof}	*	0.31	*	0.32	*	0.21	*	0.20	*	0.20	*	0.20	*	0.21	*	0.20	*	0.20
Y_{aresp}	*	0.06	*	0.07	*	0.10	*	0.12	*	0.13	*	0.15	*	0.14	*	0.15	*	0.15
Y_{em}	*	0.52	*	0.52	*	0.54	*	0.57	*	0.54	*	0.60	*	0.60	*	0.60	*	0.60
Y_{ofm}	*	0.33		0.28	*	0.21	*	0.21	*	0.20	*	0.20	*	0.21	*	0.21	*	0.21
Y_{osresp}		1.00	*	1.16	*	1.01	*	0.86	*	0.75	*	0.85	*	1.29	*	1.67	*	1.97
$k_{L}a$		280.00		280.00		280.00		280.00		280.00		280.00		280.00		280.00		280.00
pA_{max}	*	1.32	*	1.45	*	1.00	*	0.86	*	0.81	*	0.72	*	0.85	*	0.93	*	0.98
qA_{max}	*	0.57	*	0.58	*	0.95	*	0.72	*	0.65	*	0.57	*	0.65	*	0.73	*	0.68
qS_{max}	*	1.22	*	1.30	*	1.54	*	1.53	*	1.52	*	1.49	*	1.52	*	1.52	*	1.55
q_{m}		0.04		0.04		0.04		0.04		0.04		0.04		0.04		0.04		0.04

Supplementary Table S9: Parameter values after each model calibration (MC) for *Escherichia coli* K12 BW25113 *ΔgatZ*. Parameters marked with * are adjusted in the MC *

	**MC 1**		**MC 2**		**MC 3**		**MC 4**		**MC 5**		**MC 6**		**MC 7**		**MC 8**		**MC 9**	
**Parameter**	SSS	Par	SSS	Par	SSS	Par	SSS	Par	SSS	Par	SSS	Par	SSS	Par	SSS	Par	SSS	Par
K_{ap}		4.44		4.44		4.44		4.44		4.44		4.44		4.44		4.44		4.44
K_{aq}	*	0.99	*	0.96	*	0.84	*	0.97	*	0.93	*	0.92	*	0.79	*	0.71	*	0.75
K_{o}		10.00		10.00		10.00		10.00		10.00		10.00	*	13.26	*	13.21	*	14.13
K_{s}	*	0.07	*	0.09	*	0.08	*	0.10	*	0.05	*	0.06	*	0.04	*	0.03	*	0.04
K_{sq}	*	1.05	*	0.29	*	1.76	*	1.97	*	1.84	*	1.83	*	1.72	*	1.91	*	1.63
Y_{am}	*	0.41	*	0.42	*	0.43	*	0.41	*	0.46	*	0.47	*	0.46	*	0.43	*	0.44
Y_{aof}	*	0.35	*	0.31	*	0.22	*	0.20	*	0.22	*	0.22	*	0.23	*	0.21	*	0.23
Y_{aresp}	*	0.06	*	0.08	*	0.08	*	0.08	*	0.09	*	0.10	*	0.11	*	0.14	*	0.13
Y_{em}	*	0.56	*	0.51	*	0.53	*	0.53	*	0.56	*	0.58	*	0.58	*	0.60	*	0.58
Y_{ofm}		0.28		0.28	*	0.21	*	0.20	*	0.23	*	0.24	*	0.24	*	0.22	*	0.22
Y_{osresp}	*	0.76	*	1.60	*	1.14	*	0.74	*	0.88	*	1.05	*	1.43	*	1.69	*	1.89
$k_{L}a$		280.00		280.00		280.00		280.00		280.00		280.00		280.00		280.00		280.00
pA_{max}	*	1.17	*	1.54	*	1.01	*	0.71	*	0.83	*	0.86	*	1.00	*	0.94	*	1.08
qA_{max}	*	0.53	*	0.55	*	0.80	*	0.58	*	0.66	*	0.67	*	0.75	*	0.63	*	0.86
qS_{max}	*	1.21	*	1.35	*	1.35	*	1.35	*	1.34	*	1.34	*	1.35	*	1.38	*	1.39
q_{m}		0.04		0.04		0.04		0.04		0.04		0.04		0.04		0.04		0.04

Supplementary Table S10: Parameter values after each model calibration (MC) for *Escherichia coli* K12 BW25113 *ΔglcB*. Parameters marked with * are adjusted in the MC *

	**MC 1**		**MC 2**		**MC 3**		**MC 4**		**MC 5**		**MC 6**		**MC 7**		**MC 8**		**MC 9**	
**Parameter**	SSS	Par	SSS	Par	SSS	Par	SSS	Par	SSS	Par	SSS	Par	SSS	Par	SSS	Par	SSS	Par
K_{ap}		4.44		4.44		4.44		4.44		4.44		4.44		4.44		4.44		4.44
K_{aq}	*	0.98	*	0.98	*	0.86	*	0.87	*	0.99	*	0.96	*	0.92	*	0.85	*	0.70
K_{o}		10.00		10.00		10.00		10.00		10.00		10.00	*	16.53	*	13.32	*	9.57
K_{s}	*	0.07	*	0.08	*	0.08	*	0.06	*	0.03	*	0.05	*	0.04	*	0.05	*	0.03
K_{sq}		1.07	*	0.38	*	1.93	*	1.88	*	1.98	*	1.94	*	1.90	*	1.63	*	1.68
Y_{am}	*	0.42	*	0.43	*	0.40	*	0.47	*	0.41	*	0.42	*	0.41	*	0.45	*	0.42
Y_{aof}	*	0.31	*	0.39	*	0.21	*	0.21	*	0.20	*	0.21	*	0.21	*	0.23	*	0.23
Y_{aresp}	*	0.07	*	0.06	*	0.08	*	0.07	*	0.10	*	0.10	*	0.11	*	0.11	*	0.13
Y_{em}	*	0.52	*	0.51	*	0.51	*	0.53	*	0.60	*	0.59	*	0.59	*	0.58	*	0.59
Y_{ofm}		0.28		0.28		0.28	*	0.22	*	0.21	*	0.22	*	0.21	*	0.23	*	0.22
Y_{osresp}		1.00		1.00	*	1.47	*	0.86	*	0.71	*	0.77	*	0.98	*	1.14	*	1.19
$k_{L}a$		280.00		280.00		280.00		280.00		280.00		280.00		280.00		280.00		280.00
pA_{max}	*	1.21	*	1.50	*	1.05	*	0.87	*	0.60	*	0.66	*	0.69	*	0.83	*	0.87
qA_{max}	*	0.53	*	0.54	*	0.97	*	0.81	*	0.54	*	0.60	*	0.59	*	0.71	*	0.71
qS_{max}	*	1.11	*	1.09	*	1.32	*	1.31	*	1.35	*	1.36	*	1.39	*	1.38	*	1.40
q_{m}		0.04		0.04		0.04		0.04		0.04		0.04		0.04		0.04		0.04

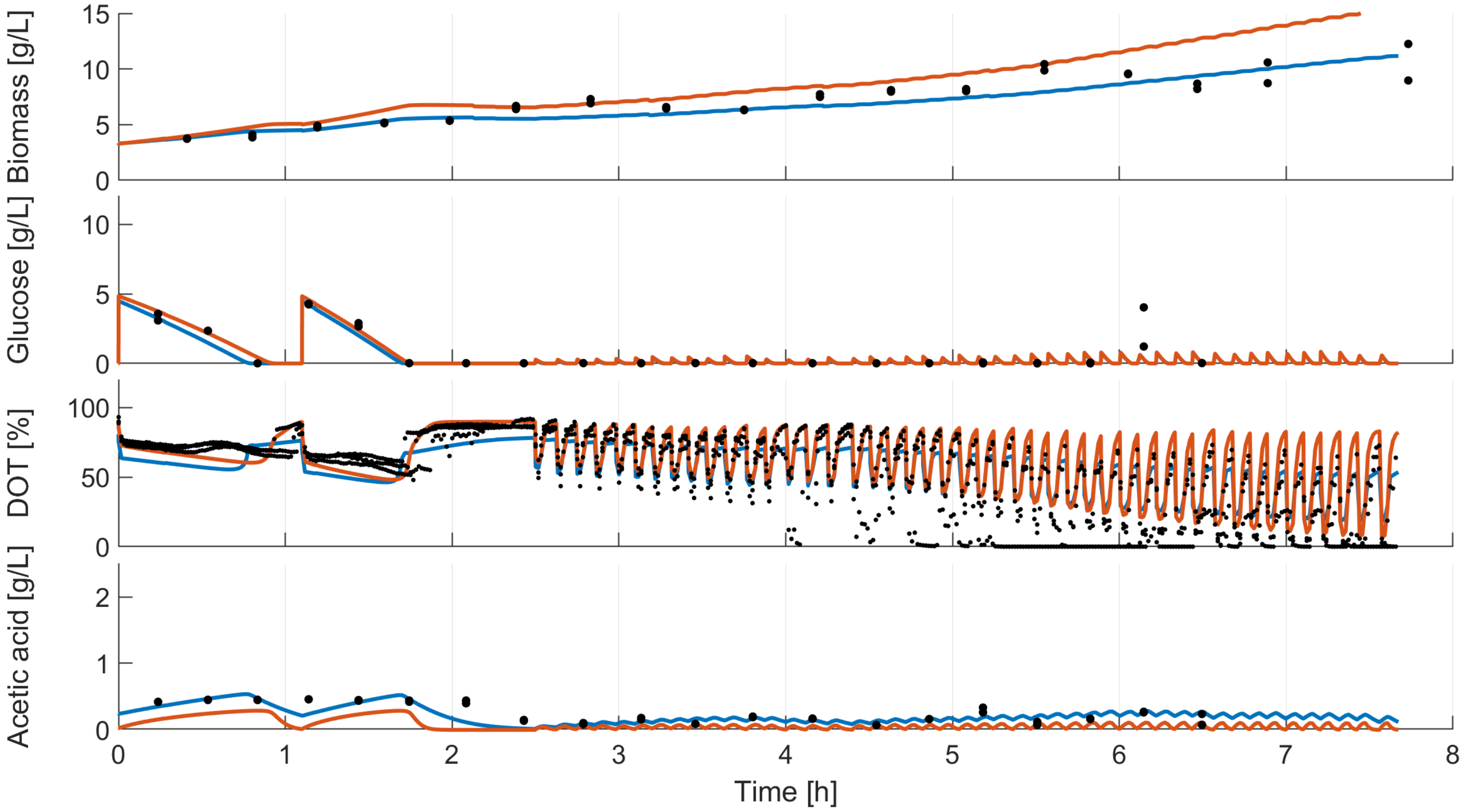

Supplementary Figure 1: Comparison of measurements (black dots) and simulation results of *E. coli* K12 W3110 with (orange line) and without model calibration (blue line). The calibrated simulation is based on the parameter set of the last model calibration after 8 hours of cultivation.
The biomass and acetic acid prediction are improved after the model calibration. Both, the trend of the cultivation and the absolute difference between measurements and simulation is improved. However, the gap between DOT and glucose measurements is slightly higher especially during the batch phase.

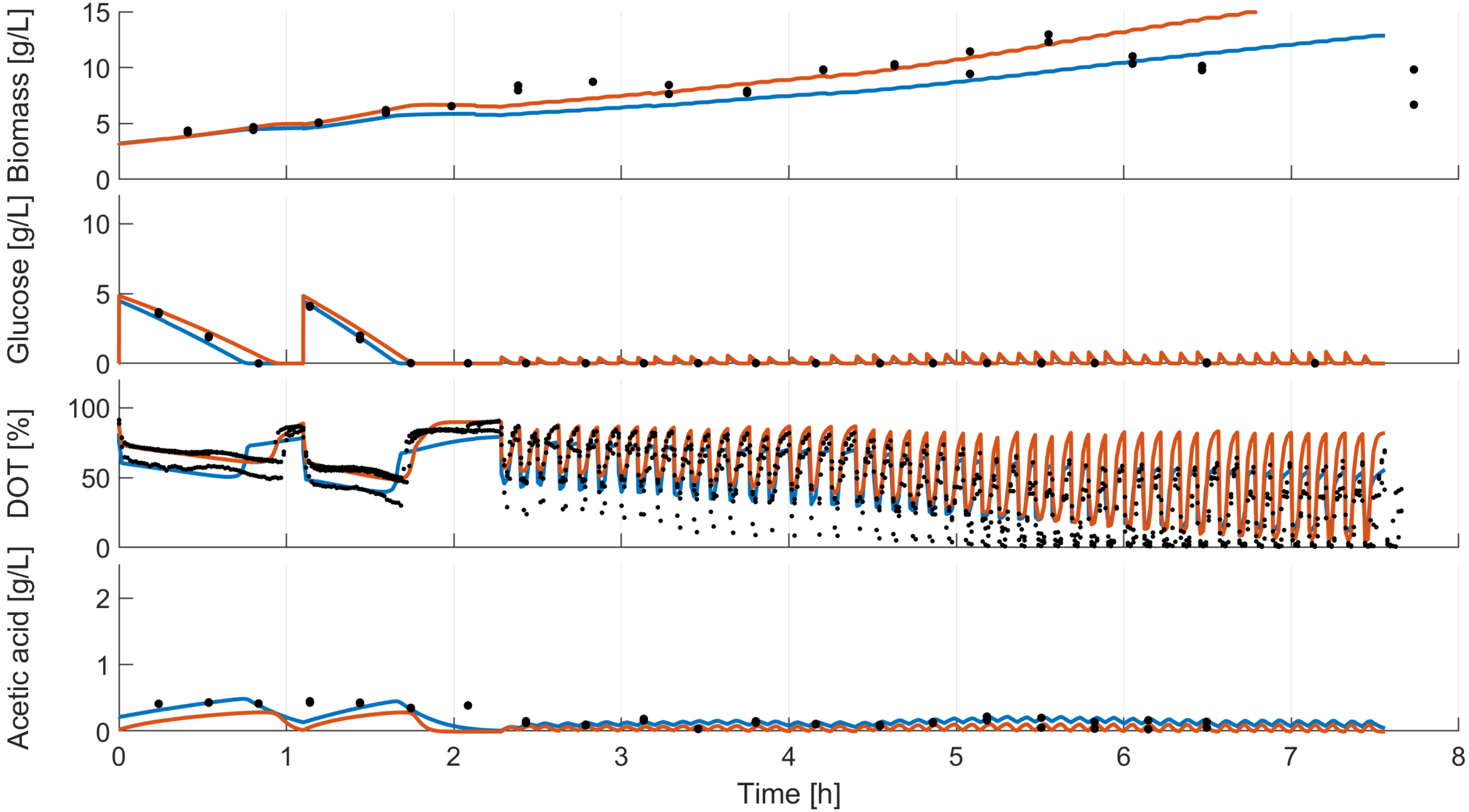

Supplementary Figure 2: Comparison of measurements (black dots) and simulation results of *E. coli* K12 BW25113 with (orange line) and without model calibration (blue line). The calibrated simulation is based on the parameter set of the last model calibration after 8 hours of cultivation.
The acetic acid prediction is slightly improved after the model calibration. However, in general the initial model seams to present the early cultivation better compared to the calibrated simulation. This is due to the difficult cultivation conditions in the last hours of cultivation. Nevertheless, the adaptive process design has been able to prevent glucose overflow and acetate accumulation by automatically reacting to the changed conditions. With a sliding window approach and automated process evaluation to detect outliers and process deviations, this issue can be addressed (see main part discussion)

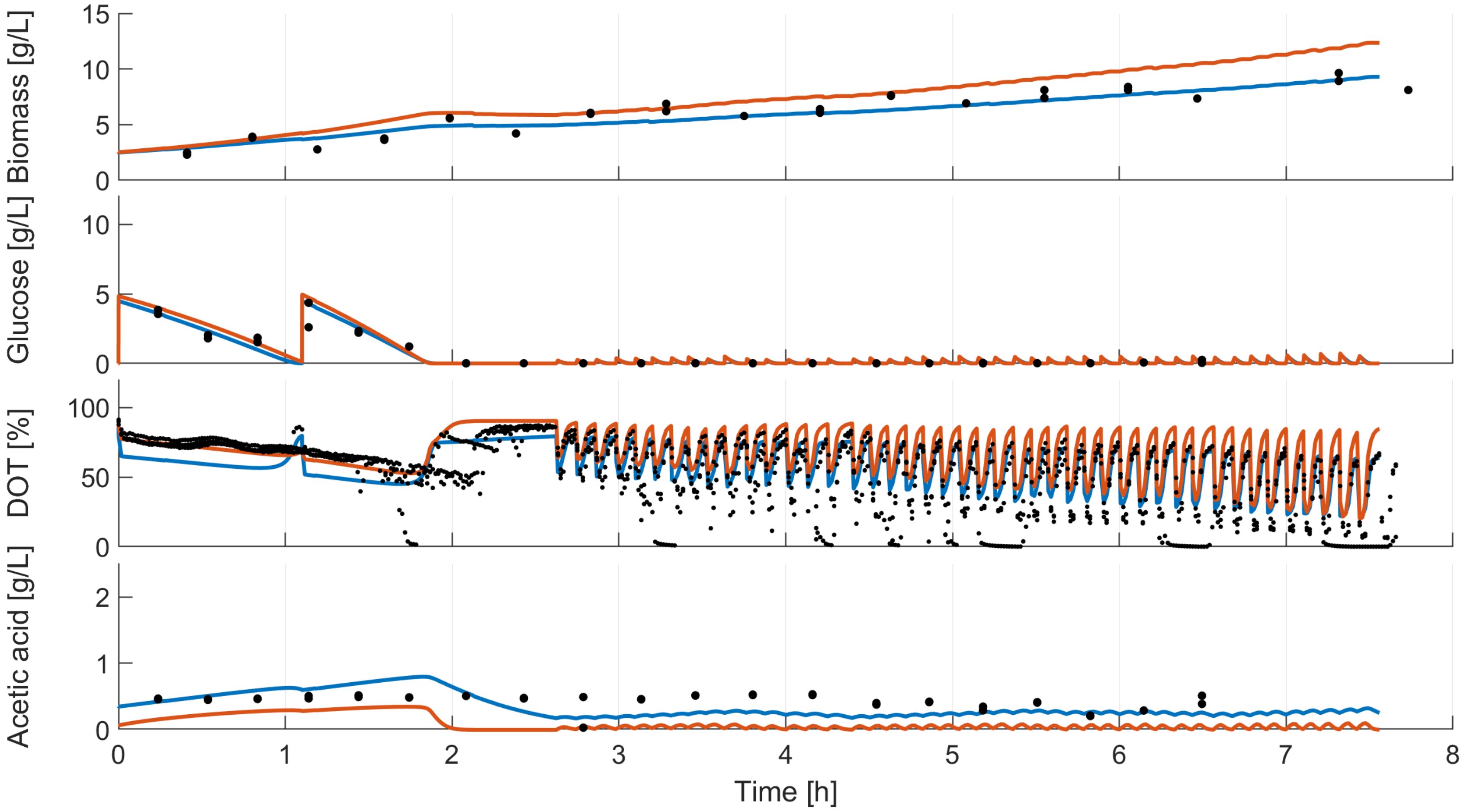

Supplementary Figure 3: Comparison of measurements (black dots) and simulation results of *E. coli* K12 BW25113 *ΔompT* with (orange line) and without model calibration (blue line). The calibrated simulation is based on the parameter set of the last model calibration after 8 hours of cultivation. The biomass and acetic acid prediction are improved after the model calibration. Both, the trend of the cultivation and the absolute difference between measurements and simulation is improved. The slight difference in the glucose prediction could not visually rated. However, based on the DOT profile the glucose uptake in the batch phase seems to be marginal overpredicted after model calibration, which is the opposed in the fed batch phase.

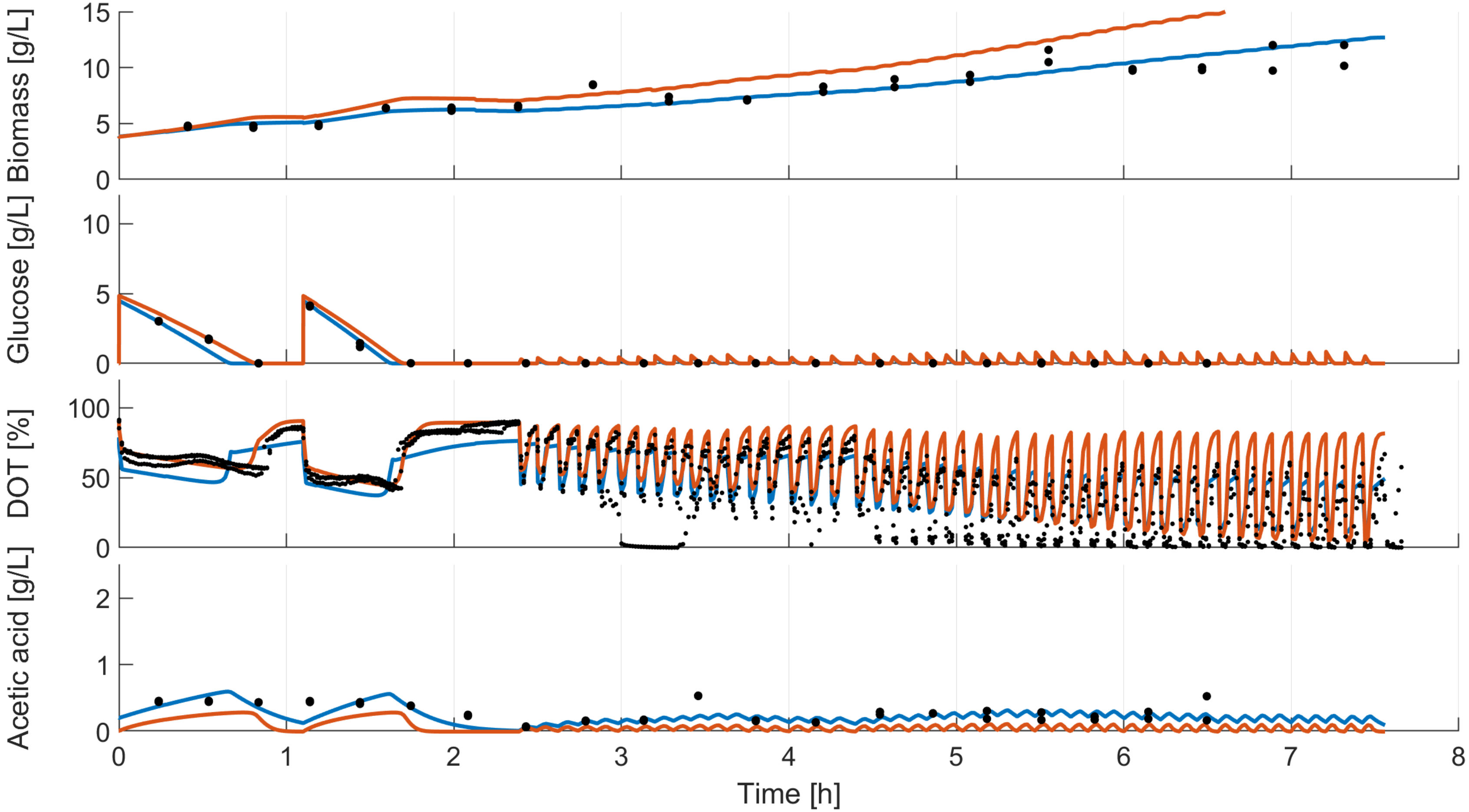

Supplementary Figure 4: Comparison of measurements (black dots) and simulation results of *E. coli* K12 BW25113 *ΔaceA* with (orange line) and without model calibration (blue line). The calibrated simulation is based on the parameter set of the last model calibration after 8 hours of cultivation. The biomass and acetic acid prediction are improved after the model calibration. Both, the trend of the cultivation and the absolute difference between measurements and simulation is improved. However, the gap between DOT and glucose measurements is slightly higher especially during the batch phase.

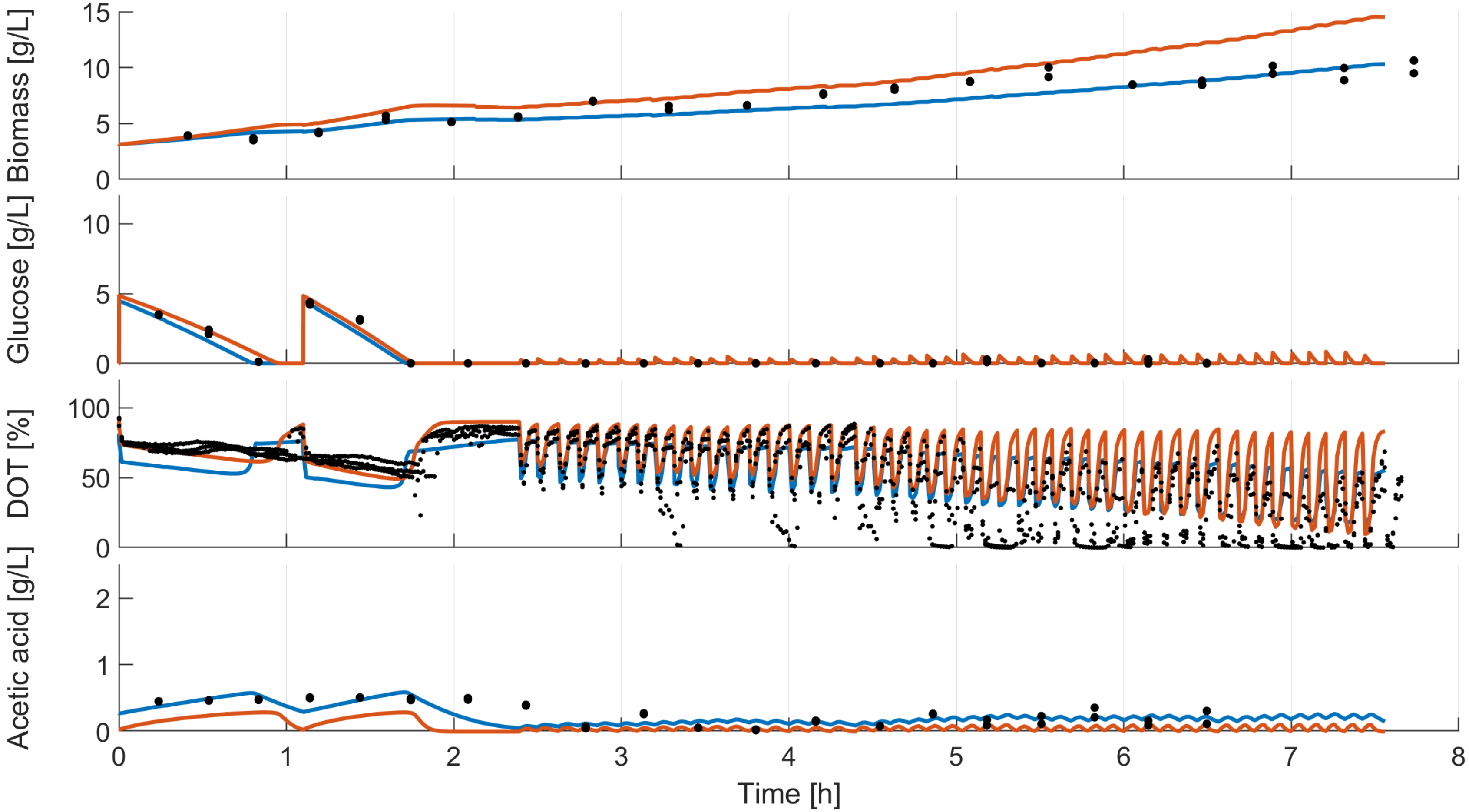

Supplementary Figure 5: Comparison of measurements (black dots) and simulation results of *E. coli* K12 BW25113 *ΔfliA* with (orange line) and without model calibration (blue line). The calibrated simulation is based on the parameter set of the last model calibration after 8 hours of cultivation. The biomass and acetic acid prediction are improved after the model calibration. Both, the trend of the cultivation and the absolute difference between measurements and simulation is improved. However, the gap between DOT and glucose measurements is slightly higher especially during the batch phase.

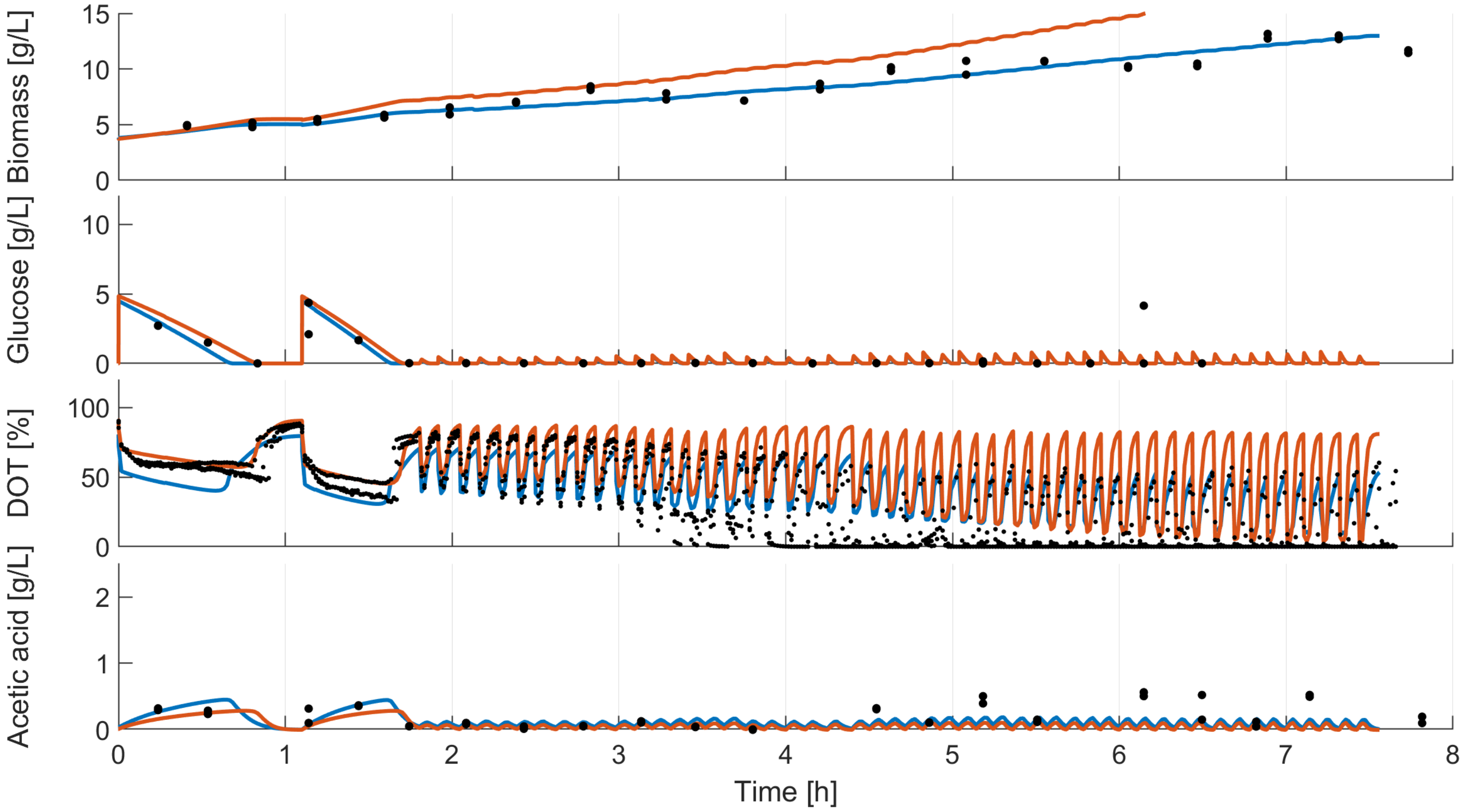

Supplementary Figure 6: Comparison of measurements (black dots) and simulation results of *E. coli* K12 BW25113 *ΔgatC* with (orange line) and without model calibration (blue line). The calibrated simulation is based on the parameter set of the last model calibration after 8 hours of cultivation. The biomass is improved after the model calibration in both, the trend of the cultivation and the absolute difference between measurements and simulation. However, the gap between DOT and glucose measurements is slightly higher especially during the batch phase.

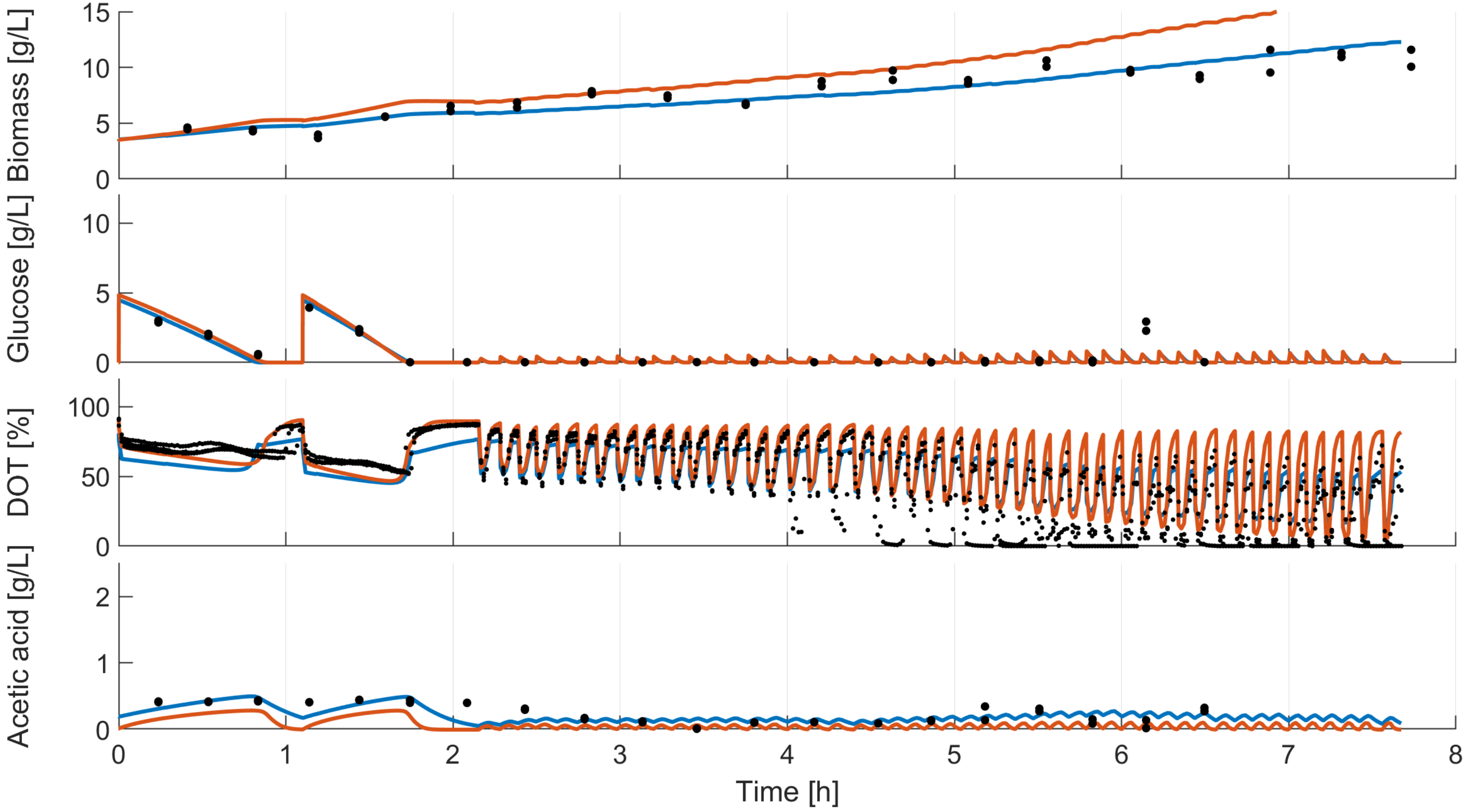

Supplementary Figure 7: Comparison of measurements (black dots) and simulation results of *E. coli* K12 BW25113 *ΔgatZ* with (orange line) and without model calibration (blue line). The calibrated simulation is based on the parameter set of the last model calibration after 8 hours of cultivation. The biomass and acetic acid prediction are improved after the model calibration. Both, the trend of the cultivation and the absolute difference between measurements and simulation is improved. The slight difference in the glucose prediction cannot be rated visually. The DOT signals followed the same trend in the initial and calibrated model. However, the oxygen uptake is overpredicted in the calibrated model.
